# Corrosion-Resistant MoO_3_/Fe_2_O_3_/MoS_2_ Heterojunctions Stabilize OH^–^ Adsorption for Efficient Light-Assisted Seawater Electrooxidation

**DOI:** 10.1021/jacs.5c04085

**Published:** 2025-06-03

**Authors:** Zhen Li, Wei Tao, Ying Wang, Xucun Ye, Yiqun Chen, Byungchan Han, Lawrence Yoon Suk Lee

**Affiliations:** † Department of Applied Biology and Chemical Technology and Research Institute for Smart Energy, 26680 The Hong Kong Polytechnic University, Hung Hom, Kowloon, Hong Kong SAR, China; ‡ Department of Chemical and Biomolecular Engineering, 26721 Yonsei University, Yonsei-ro 50, Seodaemun-gu, Seoul 03722, Republic of Korea; § Department of Chemical Engineering (Integrated Engineering), Kyung Hee University, 1732 Deogyeong-daero, Giheung-gu, Yongin-si, Gyeonggi-do 17104, Republic of Korea

## Abstract

Direct seawater electrolysis holds promise for sustainable hydrogen production, yet challenges such as severe chlorine corrosion on the anode and high energy barriers for oxygen evolution reaction (OER) limit its operational time and efficiency. Herein, we present MoO_3_/Fe_2_O_3_/MoS_2_ heterojunctions to mitigate chlorine-induced corrosion and achieve effective photoelectric synergy. The in situ leached MoO_4_^2–^ and SO_4_^2–^ inhibitors reduce Cl^–^ adsorption, thereby ensuring high OER selectivity, while the MoO_3_/Fe_2_O_3_/MoS_2_ balances the repelling effects of these inhibitors, facilitating OH^–^ adsorption and widening the overpotential gap between water oxidation and chlorine oxidation. The MoO_3_/Fe_2_O_3_/MoS_2_ catalyst outperforms its Fe_2_O_3_ counterpart in terms of lifespan, maintaining stability at 100 and 300 mA cm^–2^ for 100 and 500 h, respectively. Additionally, built-in electric fields formed at the interfaces lower interfacial resistance and extend the lifetime of photogenerated carriers by 1.47-fold, allowing for a 20.4% increase in seawater OER current density under light irradiation. Our findings offer a viable strategy for designing high-performance electrocatalysts for light-assisted seawater electrolysis.

## Graphical abstract

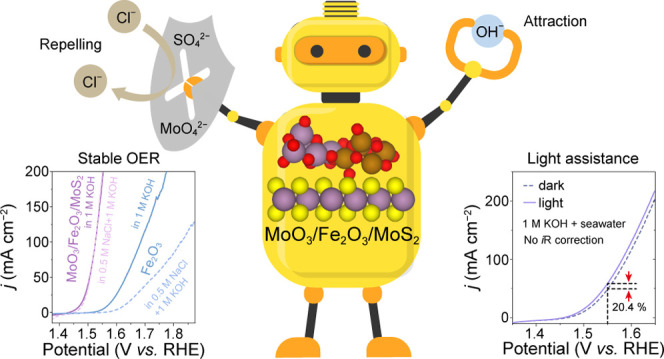

## Introduction

Green hydrogen produced through water electrolysis is pivotal for replacing fossil fuels and achieving carbon neutrality.^1,2^ However, scaling up water electrolysis raises concerns about freshwater shortage.^3–6^ As a result, seawater electrolysis has gained significant attention as a viable alternative, with seawater constituting 96.5% of the global water supply.^7^ Unlike indirect seawater electrolysis, which requires desalination pretreatment, direct seawater electrolysis simplifies the process, offering greater scalability and economic advantages.^6,8^ Nevertheless, direct seawater electrolysis faces challenges, particularly due to severe corrosion caused by the competitive chlorine evolution reaction (CER) and the high thermodynamic barrier of the oxygen evolution reaction (OER).^9,10^

Chloride anions in seawater (approximately 0.5 M) can be readily adsorbed on anodes and oxidized to Cl_2_ or ClO^–^ in acidic or alkaline seawaters, respectively, leading to severe corrosion of catalysts and compromised stability.^11,12^ For example, Fe_2_O_3_, a typical OER electrocatalyst, is prone to chlorine-induced corrosion, which limits its applicability in seawater electrolysis.^13^ Although alkalizing seawater provides a thermodynamic advantage for OER (Eanode^°^ = 1.23 V_RHE_) over CER (*E*_anode_^°^ = 1.72 V_RHE_), Cl^–^ ions can still attack electron-deficient metal sites, fostering competition between CER and the desired OER, particularly at high current densities.^14,15^ Recently, local environmental engineering has been proposed to enhance OER selectivity.^16^ Anion species such as SO_4_^2–^ and MoO_4_^2–^ introduced during the electrocatalytic process has been shown to form permselective protective layers that repel Cl^–^ through electrostatic repulsion.^17–21^ However, these anion-enriched regions may also impede the diffusion of OH^–^ ions, potentially obstructing OER.^22^ Addressing the challenge of alleviating the repelling effect of anion inhibitors on OH^–^ to ensure robust OER activity in seawater remains a significant task. Recent studies have demonstrated that constructing heterojunctions can create robust catalytic interfaces that modulate the electronic structure and OH^–^ adsorption behavior, thereby lowering the reaction energy barrier and improving electrocatalytic OER performance.^23–25^ For instance, heterojunctions composed of Fe- and Mo-based materials, such as Fe_2_O_3_/MoO_3_ and Fe_2_O_3_/P-CoMoO_4_, exhibit superior OER activity compared to their single-component counterparts.^26,27^ This enhancement is primarily attributed to the strongly coupled interfaces that facilitate charge redistribution and optimize reactant adsorption energy.

Inspired by electro-assisted photocatalysis strategies, photogenerated carrier-assisted electrocatalysis has recently emerged as a promising approach to improve water-splitting performance.^28,29^ This innovative strategy utilizes light energy to excite a photoactive component, generating carriers that promote the electrochemical reaction on the electro-active component by lowering overpotentials or increasing catalytic current densities.^30,31^ Light assistance serves as an excellent supplement for water electrolysis due to its effective utilization of clean solar energy and ease of application. While most studies on light-assisted water electrolysis have focused on broadening the absorption spectrum or modifying the electro-active component,^32–35^ the transfer of photogenerated carriers between photoactive and electro-active components in light-assisted electrocatalytic OER systems has been largely overlooked. Inefficient carrier transfer often results in rapid recombination of photogenerated electrons and holes, limiting their utilization in catalytic reactions.^36–38^ Previous reports demonstrated that introducing a built-in electric field through heterojunction formation could effectively facilitate the migration of photogenerated carriers to active sites, enabling their participation in catalytic reactions.^39–42^ For light-assisted seawater electrolysis, the primary challenge lies in simultaneously mitigating chlorine corrosion and achieving efficient photoelectric coupling.

In this study, we present an efficient and stable seawater oxidation electrocatalyst, MoO_3_/Fe_2_O_3_/MoS_2_, which exhibits exceptional OER selectivity and photoelectric coupling effect. Nanosized particles and amorphous–crystalline interfaces, induced by laser ablation, effectively increase the electrochemical surface area, enabling efficient mass diffusion. MoO_4_^2–^ and SO_4_^2–^ ions, leached during the OER activation process, contribute to repelling Cl^–^ ions in seawater. Additionally, the MoO_3_/Fe_2_O_3_/MoS_2_ heterojunctions are particularly advantageous for ensuring OH^–^ adsorption and widening the potential gap between OER and CER, thus mitigating catalyst corrosion. Furthermore, the dual built-in electric fields formed at the three-phase heterointerfaces facilitate the rapid migration of photogenerated carriers and reduce interfacial resistance, allowing more charges to participate in OER. As a result, the ternary hybrid catalyst demonstrates remarkable OER performance, achieving a current density of 10 mA cm^–2^ at a low overpotential of 267 mV in alkaline seawater and maintaining excellent stability at 100 and 300 mA cm^–2^ for 100 and 500 h, respectively. Moreover, light irradiation enhances the current density for seawater oxidation by 20.4%. The MoO_3_/Fe_2_O_3_/MoS_2_||Pt/C electrolyzer operates stably at an industry-required current density of 1 A cm^–2^ in alkaline seawater with light assistance for over 50 h. This work presents a promising strategy for developing light-assisted seawater electrolysis, paving the way for sustainable green hydrogen production.

## Results and Discussion

### Synthesis and Characterizations of Fe-L-MoS_2_

Bulk MoS_2_ powder was ablated with a pulsed laser in acetone containing Fe­(NO_3_)_3_ (0.05 M) for 25 min to produce MoS_2_ microparticles (denoted as Fe-L-MoS_2_) decorated with numerous nanoparticles (*D* = 21.5 nm, Figure 1A,B). During the laser ablation process, the MoS_2_ powder transforms into a mixture of microparticles and microplates (Figure S2a–d). The duration of laser ablation significantly influences the morphology, with microparticles gradually dominating as the ablation time is extended (Figure S2b–g). The Brunauer–Emmett–Teller (BET) adsorption–desorption isotherms and specific surface areas of samples are present in Figure S3 and Table S1, respectively. The BET surface area of Fe-L-MoS_2_ (47.35 m^2^ g^–1^) is 5.7 times greater than that of pristine MoS_2_ (8.33 m^2^ g^–1^), providing more active sites for catalytic reaction. High-resolution transmission electron microscopic (TEM) image of Fe-L-MoS_2_ (Figure 1C) reveals lattice fringes with an interplanar spacing of 2.67 Å, corresponding to the (101) plane of MoS_2_, and a distinct boundary (red dashed line) separating crystalline and amorphous regions. The amorphous regions suggest defect-rich structures, indicative of MoO_3_ and Fe_2_O_3_ formation. Scanning TEM (STEM) and the corresponding energy-dispersive X-ray spectroscopy (EDS) mapping images (Figure 1D) show uniform distributions of Mo and S across Fe-L-MoS_2_, with Fe and O enriched in the amorphous regions, supporting the presence of Fe_2_O_3_ and MoO_3_. The content of Mo and Fe elements before and after laser ablation was quantified using inductively coupled plasma optical emission spectroscopy (ICP-OES) and is summarized in Table S2. The Fe/Mo ratio increases with extended laser-ablation time, exceeding 1.0 for ablation durations longer than 25 min, indicating successful incorporation of Fe as a secondary component, rather than merely as a dopant.

**Figure 1:**
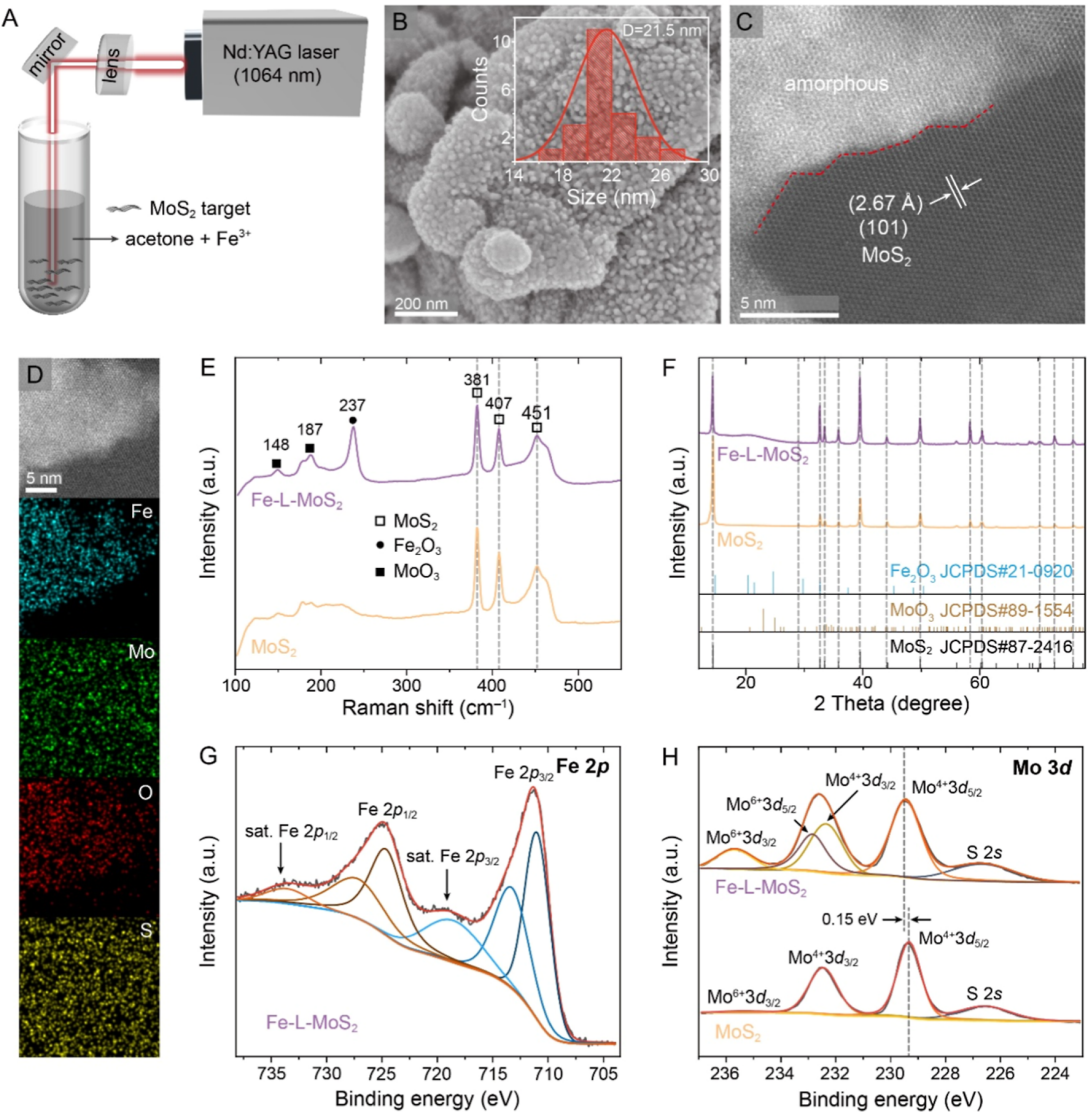
Structural characterizations of Fe-L-MoS_2_. (A) A schematic diagram illustrating the synthetic procedure of Fe-L-MoS_2_. (B) SEM (C) high-resolution TEM, and (D) STEM images with the corresponding EDS mapping images of Fe-L-MoS_2_. (E) Raman spectra and (F) XRD patterns of MoS_2_ and Fe-L-MoS_2_. XPS (G) Fe 2p spectrum of Fe-L-MoS_2_ and (H) Mo 3d spectra of MoS_2_ and Fe-L-MoS_2_.

The Raman peak observed at 237 cm^–1^ in Fe-L-MoS_2_ is attributed to the A_1g_ symmetric stretching vibrations of the Fe–O band (Figure 1E), aligning with the Raman spectrum of Fe_2_O_3_/MoS_2_ (Figure S4b), which suggests the formation of Fe_2_O_3_ after laser ablation.^43^ In addition to the characteristic Raman peaks of MoS_2_ at 381, 407, and 451 cm^–1^,^44^ two new peaks attributed to MoO_3_ are observed at 148 and 187 cm^–1^.^45^ These observations indicate the coexistence of MoO_3_ and Fe_2_O_3_ within Fe-L-MoS_2_. However, the X-ray diffraction (XRD) pattern of Fe-L-MoS_2_ (Figure 1F) is predominantly characterized by MoS_2_ peaks, with a broad peak at approximately 21° indicating the presence of amorphous Fe_2_O_3_ and MoO_3_ phases. The prominent peak at 14.5°, corresponding to the (002) plane of MoS_2_, indicates a strong preferential orientation of the MoS_2_ layers, the intrinsic interlayer stacking of MoS_2_ sheets. A possible formation mechanism involves a transient plasma plume, characterized by high temperature and high pressure, generated on the MoS_2_ target surface during pulsed laser irradiation. This plume contains oxygen and hydroxyl radicals,^46,47^ which rapidly oxidize a portion of MoS_2_ to MoO_3_ in water (Figure S5). To mitigate excessive MoS_2_ oxidation, acetone was employed as the solvent for laser ablation, as it generates reducing gases (e.g., CH_4_ and CO) that inhibit oxidation.^48^ Concurrently, Fe^3+^ ions adsorbed on negatively charged MoS_2_ (Figure S6) can easily transform into amorphous species due to the rapid cooling property of laser ablation. Figure 1G shows the X-ray photoelectron spectrum (XPS) of Fe-L-MoS_2_ in the Fe 2p region. Two distinct peaks at binding energies of 711.1 (Fe 2p_3/2_) and 724.4 eV (Fe 2p_1/2_) are observed, along with satellite peaks at 719.1 and 733.8 eV, consistent with reported Fe_2_O_3_ results.^49^ Additionally, the peak at 713.5 eV is attributed to the Fe^3+^–SO_4_^2–^ bond.^50^ Unlike pristine MoS_2_, two peaks at 531.0 and 530.1 eV, which can be assigned to Mo–O and Fe–O bonds, respectively, dominate in the O 1s spectrum of Fe-L-MoS_2_ (Figure S7b), further confirming the formation of MoO_3_ and Fe_2_O_3_.^51,52^ Similarly, in the Mo 3d spectrum of Fe-L-MoS_2_, two new peaks for Mo^6+^ 3d_5/2_ and Mo^6+^ 3d_3/2_ are observed at 232.9 and 235.7 eV, respectively (Figure 1H). Notably, the Mo^4+^ peak at 229.35 eV shifts by 0.15 eV toward higher binding energy, indicative of reduced electronic density around Mo atoms and suggesting strong interaction among the three components.^53^ The Mo^6+^/Mo^4+^ atomic ratios of Fe-L-MoS_2_ samples synthesized using different laser ablation times are estimated (Figure S8) and summarized in Table S3. This ratio increases with the laser ablation time, reaching a value of 0.48 at 25 min-ablation. Furthermore, the S–Fe bond is identified at 162.7 eV in the S 2p spectrum (Figure S7c),^54,55^ consistent with the Fe 2p results and indicating the interaction between Fe_2_O_3_ and MoS_2_.

### Electrocatalytic Performance in Alkaline Freshwater and Seawater

The OER activity of the as-prepared samples was evaluated in O_2_-saturated 1 M KOH using a standard three-electrode cell. Figure 2A compares linear sweep voltammograms (LSVs) collected at a scan rate of 2 mV s^–1^. Fe-L-MoS_2_ requires overpotentials of 241 and 296 mV to reach current densities of 10 and 100 mA cm^–2^, respectively (Figure S9a), which are significantly lower compared to Fe_2_O_3_/MoS_2_ (282 and 343 mV), pristine MoS_2_ (307 and 406 mV), and Fe_2_O_3_ (339 and 455 mV). The Tafel plots derived from the polarization curves reveal that Fe-L-MoS_2_ exhibits the lowest Tafel slope (48.9 mV dec^–1^) among all samples investigated (Figure 2B), indicating its rapid reaction kinetics. When comparing OER overpotential at 10 mA cm^–2^ and Tafel slope, Fe-L-MoS_2_ outperforms or matches other benchmark MoS_2_- and Fe_2_O_3_-based catalysts (Figure S9b and Table S4). Moreover, Fe-L-MoS_2_ demonstrates a high turnover frequency (TOF) value of 0.154 s^–1^ (Figure 2C), approximately 54.6 times that of Fe_2_O_3_ (0.003 s^–1^), highlighting the substantial enhancement of intrinsic catalytic activity achieved through the formation of amorphous/crystalline heterojunctions. The OER performance of Fe-L-MoS_2_ can be optimized by adjusting the Fe^3+^ concentration and laser-ablation time (Figure S10). The lowest OER overpotential is achieved with a sample synthesized using 0.05 M Fe­(NO_3_)_3_ and 25 min of ablation, yielding a 34.2% Fe_2_O_3_ content and a Mo^6+^/Mo^4+^ ratio of *ca*. 0.48 (Figure S11). Acetone’s low polarity enhances Fe_2_O_3_ deposition on the sample surface, thereby improving its OER activity (Figure S12). Excessive laser ablation or Fe^3+^ usage decreases OER performance due to increased charge transfer resistance (Figure S13), likely caused by the excessive formation of Fe_2_O_3_ and MoO_3_ (Table S3).

**Figure 2:**
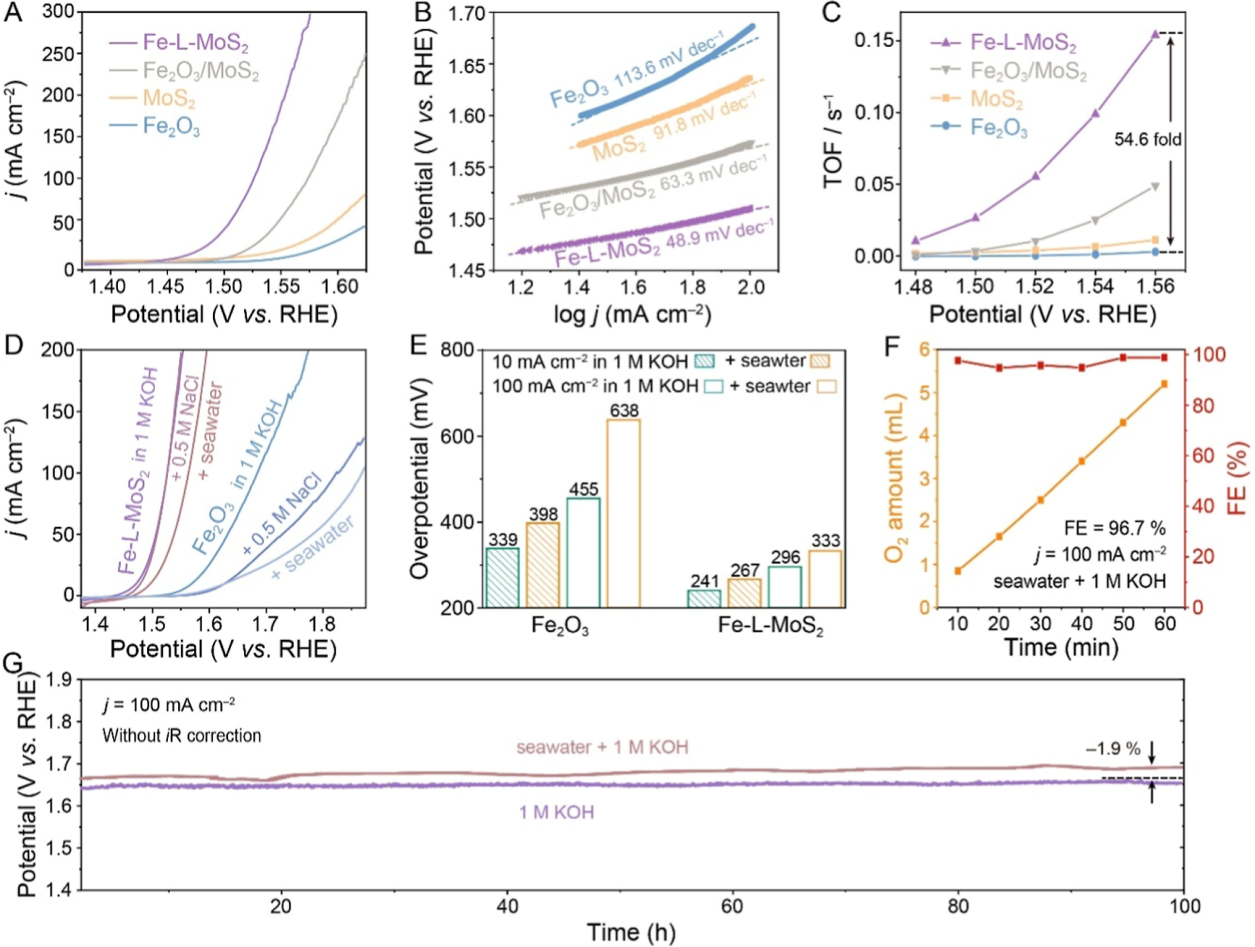
OER performance in alkaline freshwater and seawater. (A) LSV curves, (B) the corresponding Tafel slopes, and (C) TOF of Fe_2_O_3_, MoS_2_, Fe_2_O_3_/MoS_2_, and Fe-L-MoS_2_. (D) LSV curves of Fe-L-MoS_2_ and Fe_2_O_3_ in alkaline saline water and seawater. (E) Comparison of OER overpotentials of Fe-L-MoS_2_ and Fe_2_O_3_ in various electrolytes. (F) FE (red squares) of Fe-L-MoS_2_ measured at 100 mA cm^–2^ in alkaline seawater. Yellow squares indicate the amount of O_2_ generated during the FE test. (G) Chronopotentiograms of Fe-L-MoS_2_ in alkaline freshwater and seawater.

The OER activity of Fe-L-MoS_2_ was further evaluated in alkaline saline (0.5 M NaCl + 1 M KOH) and alkaline natural seawater (seawater +1 M KOH) electrolytes to explore its potential for seawater electrolysis application. As shown in Figure 2D, Fe-L-MoS_2_ exhibits superior OER performance with minimal activity decline in saline water electrolytes, indicating that the impact of highly concentrated Cl^–^ ions on its catalytic activity is not significant. However, a noticeable deterioration in OER performance is observed in the alkaline seawater, which can be attributed to the presence of bacteria, microbes, and insoluble precipitates formed during the seawater OER process.^56^ In alkaline seawater, the Fe-L-MoS_2_ electrode requires only 267 and 333 mV to reach 10 and 100 mA cm^–2^, respectively (Figure 2E), which are considerably lower than those of Fe_2_O_3_ (398 and 638 mV) under the same conditions. Notably, the electrode achieves a high average Faradaic efficiency (FE) of 96.7% at a large current density of 100 mA cm^–2^ in alkaline seawater (Figure 2F). Iodide titration was engaged to detect the generation of reactive chlorine species.^57^ No characteristic absorption peak of hypochlorite ions is observed in the electrolyte after the FE test (Figure S14b), indicating the catalyst’s high selectivity for OER over the hypochlorite formation reaction. Operational stability is another crucial parameter for electrocatalysts. To assess the electrocatalytic durability of Fe-L-MoS_2_ during OER, long-term chronopotentiometry was conducted in both alkaline freshwater and seawater. Remarkably, Fe-L-MoS_2_ demonstrates excellent stability at a current density of 100 mA cm^–2^ in both alkaline electrolytes over a 100 h period (Figure 2G), with only a slight increase (1.89%) in the required potential for seawater OER.

### Exploration of OER and Anticorrosion Mechanisms

Reconstruction is a common occurrence during the OER activation process. After OER activation, the XRD pattern of Fe-L-MoS_2_, excluding the Ni foam substrate, continues to be dominated by the MoS_2_ phase (Figure S15a), indicating that no new species are formed during activation. In the Raman spectra (Figure S15b), all peaks corresponding to MoS_2_, Fe_2_O_3_, and MoO_3_ remain at the same positions. However, the intensity of the MoS_2_ peak is slightly reduced compared to that of Fe_2_O_3_, suggesting a minor loss of MoS_2_. Postactivation, Fe-L-MoS_2_ retains its morphology, characterized by microparticles decorated with nanoparticles (Figure S16). High-resolution TEM images (Figure S17a) show an interplanar spacing of 2.7 Å, corresponding to the (100) plane of MoS_2_, along with several amorphous regions. EDS mapping images (Figure S17b) confirm the uniform distribution of Mo, Fe, O, and S elements across the activated sample. These results collectively indicate that Fe-L-MoS_2_ largely maintains its structural integrity after OER activation. Notably, the presence of MoO_4_^2–^ and SO_4_^2–^ is observed in the XPS spectra (Figure S18) following OER activation, which may play a significant role in enhancing anticorrosion properties.

Density functional theory (DFT) calculations were conducted to gain insights into how heterojunctions enhance both the water oxidation activity and anticorrosion properties of catalysts. Models of amorphous Fe_2_O_3_, amorphous/crystalline Fe_2_O_3_/MoS_2_, and MoO_3_/Fe_2_O_3_/MoS_2_ heterojunctions were constructed using ab initio molecular dynamics simulations, and their crystal structures are provided in Figure S19. The charge redistribution across the three-phase heterojunction in Fe-L-MoS_2_ was quantitatively analyzed using planar-averaged differential charge density (Δρ). Positive and negative values indicate charge accumulation and depletion, respectively. Figure 3A shows the Δρ profile along the *z*-direction (perpendicular to the interface), with charge accumulation and depletion depicted in yellow and cyan regions. Along the *z*-axis, charge depletion is observed at the interface between the MoS_2_ and the two amorphous oxides, while charge accumulation occurs where the two amorphous oxides are located. This result suggests charge transfer from the MoS_2_ to the amorphous oxides upon heterointerface formation.

**Figure 3:**
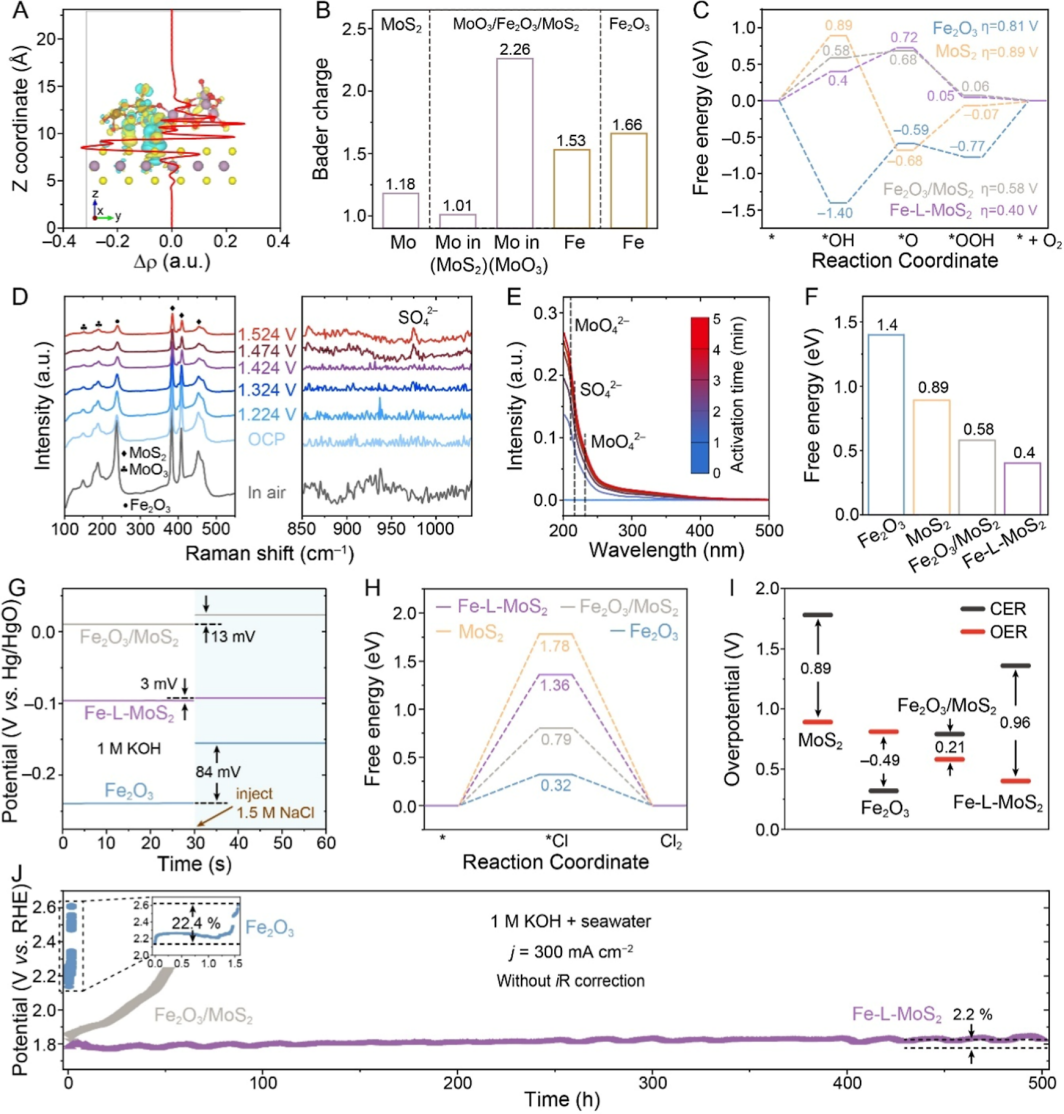
OER and corrosion-resistant mechanisms. (A) Charge density difference and planar average charge density difference (red line) along the *z*-direction of the MoO_3_/Fe_2_O_3_/MoS_2_ heterojunction in Fe-L-MoS_2_. (B) Charges on Mo and Fe in Fe_2_O_3_, MoS_2_, and Fe-L-MoS_2_. (C) Gibbs free energy diagrams for OER. (D) In situ Raman spectrum of Fe-L-MoS_2_. (E) Quasi-in situ UV–vis spectra for MoO_4_^2–^ and SO_4_^2–^ detection. (F) Free energy change on E_*OH_–*E*_slab_, (G) OCP profiles (H) Gibbs free energy diagrams for CER, and (I) the corresponding overpotential gaps between OER and CER for Fe_2_O_3_, MoS_2_, Fe_2_O_3_/MoS_2_, and Fe-L-MoS_2_. (J) Chronopotentiograms of Fe_2_O_3_, Fe_2_O_3_/MoS_2_, and Fe-L-MoS_2_ in seawater +1 M KOH.

Bader charge distributions at the binary and ternary heterointerfaces are given in Figure S20. The net charge at the Mo site in the MoS_2_ phase of the Fe_2_O_3_/MoS_2_ heterointerface is higher than in the pristine MoS_2_ phase (Figure S21), while the charge at the Fe site in the Fe_2_O_3_ phase is lower than in single-phase Fe_2_O_3_, indicating charge transfer from MoS_2_ to Fe_2_O_3_. Upon incorporating a third phase, MoO_3_, to form the MoO_3_/Fe_2_O_3_/MoS_2_ ternary heterointerface, the Mo site in MoO_3_ exhibits a higher Bader charge compared to single-phase MoS_2_ (Figure 3B), while the Fe site has a lower Bader charge compared to single-phase Fe_2_O_3_. This confirms charge transfers from MoO_3_ to Fe_2_O_3_ in the ternary heterojunction, suggesting Fe as the reactive site. The change in Bader charge at Fe and Mo sites in MoO_3_/Fe_2_O_3_/MoS_2_ compared to the binary hybrid can be ascribed to the strong interaction in the ternary heterointerface.

Electrochemical impedance spectroscopy (EIS) was employed to probe the charge transfer process, and the Nyquist plots are provided in Figure S22 and Table S5. The catalyst resistance (*R*_2_) for MoS_2_ (0.61 Ω), Fe_2_O_3_ (3.30 Ω), and Fe_2_O_3_/MoS_2_ (1.26 Ω) is considerably reduced to 0.24 Ω in Fe-L-MoS_2_, demonstrating the effective synergistic effects of three-phase heterointerfaces in lowering the charge transfer barrier in electrocatalysts. The solid–liquid interface resistance (*R*_ct_) also decreases from 114.50 Ω (Fe_2_O_3_) and 74.67 Ω (MoS_2_) to 2.0 Ω in Fe-L-MoS_2_, indicating facilitated charge exchange between the electrode and the electrolyte, as well as improved OER kinetics.^58^ The formation of heterointerfaces promotes charge redistribution within the space charge layer at the electrode–solution interface.

Mott–Schottky analysis was performed to assess charge concentration by measuring capacitance across a range of applied potentials.^59^ The Mott–Schottky plot for Fe-L-MoS_2_ reveals a carrier density of 5.39 × 10^21^ cm^–3^, which is approximately 14.9, 18.7, and 54.2 times greater than that of the Fe_2_O_3_/MoS_2_ hybrid (3.61 × 10^20^ cm^–3^), pristine MoS_2_ (2.88 × 10^20^ cm^–3^), and Fe_2_O_3_ (9.95 × 10^19^ cm^–3^), respectively (Figure S23). This enhanced carrier density indicates that the Fe-L-MoS_2_ interface provides more mobile charges available for catalytic reactions, potentially contributing to improved catalytic performance.

DFT calculations were also employed to determine the free energy diagrams for each elementary step during the OER (Figures S24–S27). The OER free energy diagram in Figure 3C reveals that for Fe_2_O_3_, the rate-determining step (RDS) is the deprotonation and electron transfer from *OH to form *O (the second step). In contrast, for both Fe_2_O_3_/MoS_2_ and Fe-L-MoS_2_, the RDS shifts to the *OH adsorption (the first step). At the same time, the overall OER free energy decreases significantly from 0.81, 0.89, and 0.58 eV for Fe_2_O_3_, MoS_2_, and Fe_2_O_3_/MoS_2_, respectively, to 0.40 eV for Fe-L-MoS_2_, highlighting the beneficial effects of heterointerfaces on OER catalysis.

To further investigate the impact of interfaces on OER thermodynamics, Arrhenius plots of Fe_2_O_3_, MoS_2_, and Fe-L-MoS_2_ were obtained by measuring LSVs at various temperatures (Figures S28 and S29). The activation energy (*E*_a_), extracted from the slope of the Arrhenius plot, is the lowest for Fe-L-MoS_2_ (23.5 kJ mol^–1^), followed by Fe_2_O_3_/MoS_2_ (37.4 kJ mol^–1^), MoS_2_ (41.3 kJ mol^–1^), and Fe_2_O_3_ (53.5 kJ mol^–1^). This reduction in *E*_a_ indicates a significant decrease in the kinetic barrier for electrocatalytic water oxidation at the heterojunctions. Electrochemical surface areas (ECSAs) of the as-prepared catalysts were also determined using electrochemical double-layer capacitance (*C*_dl_) from cyclic voltammograms (Figures S30 and S31). Notably, Fe-L-MoS_2_ exhibits an ECSA of 4.88 cm^2^ mg^–1^, which is smaller than that of pristine MoS_2_ (6.82 cm^2^ mg^–1^). This indicates there is no clear positive correlation between specific surface area and specific capacitance. The large and complete layered structure of MoS_2_ is more conducive to promoting the reversible insertion and extraction of ions in the electrolyte, thereby exhibiting superior capacitance. After normalizing the polarization curves by ECSA (Figure S32), Fe-L-MoS_2_ maintains superior OER activity compared to its counterparts. This suggests that its outstanding performance stems from enhanced intrinsic catalytic activity facilitated by the three-phase interfaces.

In situ Raman spectroscopy was employed to monitor surface species as the applied potential varies in real time. Figure 3D presents the in situ Raman spectra of Fe-L-MoS_2_ collected between the open-circuit potential (OCP) and an applied potential of 1.524 V. Characteristic peaks corresponding to MoO_3_, Fe_2_O_3_, and MoS_2_ are evident at the OCP, with decreasing intensity as applied potential increases. Notably, a new peak at 975 cm^–1^, attributed to SO_4_^2–^, appears at 1.474 V,^60^ indicating the oxidation of MoS_2_ during the OER process. Quasi-in situ UV–vis spectroscopy further confirms the release of MoO_4_^2–^ and SO_4_^2–^ anions (Figure S33). Upon applying 2.5 V to Fe-L-MoS_2_ in a two-electrode system, absorption peaks for MoO_4_^2–^ (211 and 232 nm) and SO_4_^2–^ (216 nm) appear after 1 min (Figure 3E). These peaks intensify with prolonged oxidation time, substantiating the release of MoO_4_^2–^ and SO_4_^2–^ from MoS_2_.

The presence of MoO_4_^2–^ and SO_4_^2–^ anions around Fe-L-MoS_2_, formed under anodic potential, can repel Cl^–^ through electrostatic repulsion, thereby mitigating corrosion during seawater oxidation.^19^ Additionally, Fe-L-MoS_2_ exhibits the smallest energy gap between E_*OH_ and *E*_slab_ (Figure 3F), indicating that the heterojunction enhances OH^–^ adsorption, counteracting the electrostatic repulsion from anions. The strong hydrogen bonding between OH^–^ and MoS_2_ further prevents electrostatic repulsion during OH^–^ diffusion, ensuring rapid OER kinetics.^61^ This is supported by the OCP measurements, which reflect Cl^–^ adsorption on the Helmholtz layer; a greater influence of the catalyst surface on Cl^–^ results in a more profound shift in OCP upon introducing Cl^–^.^62,63^ Typically, the exchange of adsorbates and ions within the Helmholtz layer disrupts the electrode potential equilibrium. Upon adding 1.5 M NaCl to the electrolyte, a significant increase in OCP of 84 mV is observed for Fe_2_O_3_ compared to Fe_2_O_3_/MoS_2_ (13 mV, Figure 3G). This change further decreases to 3 mV for Fe-L-MoS_2_, demonstrating that the coupling effect of MoO_4_^2–^/SO_4_^2–^ ions and MoO_3_/Fe_2_O_3_/MoS_2_ heterojunctions in Fe-L-MoS_2_ can further stabilize the Helmholtz layer. In other words, the heterojunction balances the electrostatic effects of anion inhibitors on OH^–^ and stabilizes OH^–^ adsorption. XPS analysis post-OER activation in saline electrolyte (0.5 M NaCl + 1 M KOH) reveals a Cl 2p peak at 198.3 eV for Fe_2_O_3_ (Figure S34a), confirming Cl^–^ adsorption,^64^ whereas no Cl^–^ signal is detected for Fe-L-MoS_2_ (Figure S34b), underscoring its superior Cl^–^-repelling property. Although the overpotential gap at 100 mA cm^–2^ between freshwater and saline water is reduced from 139 mV on Fe_2_O_3_ to 10 mV on Fe_2_O_3_/MoS_2_ (Figure S35b), the OER performance in saline water remains inferior to that in freshwater due to the repelling effect of leached MoO_4_^2–^ and SO_4_^2–^ on OH^–^ adsorption. Notably, Fe-L-MoS_2_ exhibits similar LSV curves in both electrolytes (Figure S35a). The overpotential gap is further reduced to 0 mV with Fe-L-MoS_2_, indicating that the MoO_3_/Fe_2_O_3_/MoS_2_ heterojunction effectively balances the repelling influence of anion inhibitors on OH^–^, thereby ensuring OH^–^ adsorption for efficient OER catalysis. Considering that adsorbed Cl^–^ ions on active sites can hinder the oxidation of OH^–^, thereby limiting OER catalysis, the OER activity, particularly at low current densities, is positively correlated with the number of effective active sites. As shown in Figure S36a, Fe_2_O_3_ exhibits a 16.3% decrease in current density at the onset potential when the electrolyte is switched to 1 M KOH + 0.5 M NaCl. This suggests that 16.3% of the active sites are poisoned by Cl^–^ ions. In contrast, Fe-L-MoS_2_ shows no decrease in current density under the same conditions (Figure S36b), indicating its superior Cl^–^-repelling properties.

The CER process in Fe-L-MoS_2_ was investigated using a two-step Volmer–Heyrovsky mechanism, encompassing Cl^–^ adsorption and subsequent molecular Cl_2_ release (Figures S37–S40). The Gibbs free energy changes for each elementary step of the CER are presented in Figure 3H. The free energy diagram indicates that Fe-L-MoS_2_ requires substantially higher energy (1.36 eV) for CER compared to Fe_2_O_3_ (0.32 eV), signifying that the heterojunction effectively suppresses CER, thereby enhancing the corrosion resistance of the electrode. MoS_2_ exhibits the greatest free energy for CER due to the unfavorable Cl^–^ adsorption on S sites. Additionally, the thermodynamic OER selectivity can be described by the overpotential gap between OER and CER (η_CER_ – η_OER_), where a large gap indicates a higher selectivity of OER. The overpotential gap for Fe_2_O_3_ is determined to be −0.49 V (Figure 3I), suggesting that CER is more favorable than OER. In contrast, positive overpotential gap values for MoS_2_, Fe_2_O_3_/MoS_2_, and Fe-L-MoS_2_ indicate that OER dominates the anodic reaction, with Fe-L-MoS_2_ exhibiting the largest gap of 0.96 V for enhanced OER selectivity.

Long-term seawater electrolysis at a high current density of 300 mA cm^–2^ was conducted on Fe_2_O_3_, Fe_2_O_3_/MoS_2_, and Fe-L-MoS_2_ (Figure 3J). Fe-L-MoS_2_ maintains relatively stable activity over 500 h, while Fe_2_O_3_ and Fe_2_O_3_/MoS_2_ rapidly lose activity within 1.5 and 53 h, respectively. Although Fe_2_O_3_/MoS_2_ exhibits a slight increase (2.2%) in the potential required to sustain 300 mA cm^–2^ for seawater OER, this is significantly less than the 22.4% increase observed for Fe_2_O_3_ within just 1.5 h, further confirming the excellent corrosion resistance of Fe-L-MoS_2_. The voltage degradation rate (*D*_U_) is calculated using $D_{U}=\frac{{\mathrm{U̅}}_{2}-{\mathrm{U̅}}_{1}}{t}$ , where *U̅*_1_ and *U̅*_2_ are the average potentials during the initial and final 10% of the test duration (*t*), respectively, to mitigate fluctuations in long-term operation.^65^ In a 500 h chronopotentiometry test at 300 mA cm^–2^, Fe-L-MoS_2_ exhibits a *D*_U_ of 0.078 mV h^–1^, significantly lower than that of Fe_2_O_3_ (0.319 mV h^–1^), demonstrating its superior stability for seawater electrolysis. Notably, no characteristic absorption peak of hypochlorite ions is observed in the electrolyte after the 500 h catalytic operation (Figure S41), indicating the high selectivity of Fe-L-MoS_2_ for the OER over the hypochlorite formation reaction, even at higher current densities. Corrosion polarization curves obtained in natural seawater show that Fe-L-MoS_2_ has a significantly lower corrosion current density (0.101 mA cm^–2^) and a higher corrosion potential (−0.28 V) compared to Fe_2_O_3_ (1.101 mA cm^–2^ and −0.37 V), indicating stronger corrosion resistance in seawater (Figures S42 and S43).^66^ Notably, the Raman peaks for Mo–O, Fe–O, and Mo–S vibrations are observed after OER activation and a 100 h stability test in alkaline seawater (Figure S44), suggesting the integrity of the three-phase structure. However, the intensity of the Mo–S mode has diminished relative to the Fe–O mode after OER activation, likely due to the oxidation of MoS_2_. The nanoparticles on Fe-L-MoS_2_ are preserved during seawater electrolysis (Figure S45a), confirming its high structure stability. Additionally, the presence of Ca is detected, attributed to the formation of insoluble Ca­(OH)_2_ on the catalyst’s surface during OER (Figure S45b), which may explain the observed performance decay in the alkaline seawater electrolysis.

### Light-Assisted OER Performance and Mechanism

The light-assisted electrochemical OER performance of Fe-L-MoS_2_ was further evaluated under UV–Vis irradiation in alkaline seawater. At an applied potential of 1.550 V (vs RHE), the current density increases by 20.4% (Figure 4A), with a phototo-current efficiency (PCE) of 10.66%. The Tafel slope decreases from 48.9 to 41.9 mV dec^–1^ under irradiation (Figure 4B), indicating accelerated OER kinetics. Compared to other light-responsive electrocatalysts, Fe-L-MoS_2_ exhibits superior light-assisted OER activity in 1 M KOH and seawater (Figure S46 and Table S6). Although designing electrolytic cells and catalysts for light-assisted water electrolysis poses challenges, this approach holds significant promise for seawater electrolysis by leveraging solar energy to reduce costs associated with long-distance power transmission, on-site power generation, and maintenance, making it a cost-effective solution in specific contexts. Encouraged by these promising results, a two-electrode electrolyzer was assembled using Fe-L-MoS_2_ and 20% Pt/C as the anode and cathode, respectively, to evaluate light-assisted seawater splitting performance at large current densities. The Fe-L-MoS_2_||Pt/C seawater electrolyzer delivers a current density of 0.5 A cm^–2^ at a cell voltage of 2.476 V (Figure 4C), significantly lower than the benchmark RuO_2_||Pt/C system (2.718 V). Moreover, during a 170 h chronopotentiometry test at 0.2 A cm^–2^ (Figure 4D), the cell potential decreases under light irradiation and remains lower until the light is turned off, demonstrating stable light-assisted OER catalysis of Fe-L-MoS_2_ in seawater. Postlight-assisted *U*–*t* test characterizations confirm the robustness of the MoO_3_/Fe_2_O_3_/MoS_2_ heterojunction. XPS analysis reveals persistent MoO_4_^2–^ and SO_4_^2–^ anions (Figure S47), while TEM images show an intact crystalline/amorphous interface (Figure S48), indicating strong anticorrosion properties of the three-phase heterojunction.

**Figure 4:**
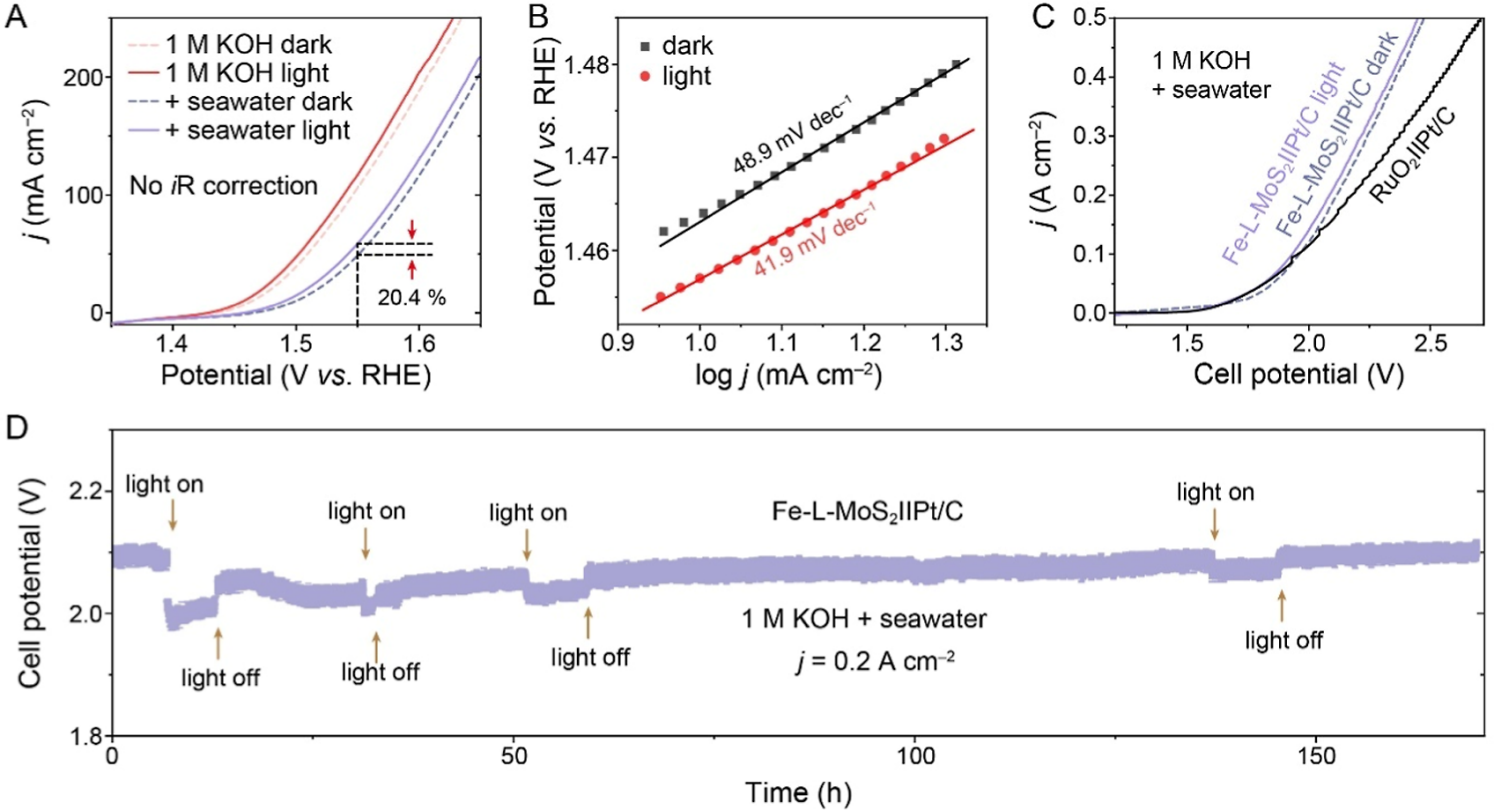
Light-assisted OER and seawater electrolysis performance. (A) LSV curves of Fe-L-MoS_2_ in the dark and under light irradiation in various electrolytes and (B) the corresponding Tafel plots. (C) LSV curves measured using two-electrode systems of Fe-L-MoS_2_||Pt/C and RuO_2_||Pt/C. (D) Chronopotentiogram of Fe-L-MoS_2_||Pt/C alkaline seawater electrolyzer operated at 0.2 A cm^–2^.

To better understand the light-assisted OER mechanism, the band structures of MoS_2_, Fe_2_O_3_, and MoO_3_ before and after contact are illustrated in Figure 5A. The work functions (Φ) of MoS_2_, Fe_2_O_3_, and Fe-L-MoS_2_ were determined using ultraviolet photoelectron spectroscopy (UPS, Figure S49).^67^ The work function of MoS_2_ (5.62 eV) is lower than that of Fe_2_O_3_ (6.60 eV), indicating that electrons spontaneously migrate from MoS_2_ to Fe_2_O_3_ upon interface formation, thereby narrowing their Fermi level (*E*_F_) gap. This electron transfer establishes a built-in electric field I, directed from MoS_2_ to Fe_2_O_3_, resulting in charge redistribution at the interface. Consequently, Fe_2_O_3_ becomes negatively charged while the surface of MoS_2_ becomes electrophilic. The positively charged MoS_2_ can attract electron-rich OH^–^, which is expected to enhance catalytic kinetics, while the negatively charged Fe_2_O_3_ improves corrosion resistance by repelling Cl^–^. A second built-in electric field II, directed from MoO_3_ to Fe_2_O_3_, forms at the Fe_2_O_3_/MoO_3_ interface. Under light irradiation, electrons are excited from valence band maximum (VBM) to conduction band minimum (CBM), leaving photogenerated holes in the VBM of those three components. The built-in electric fields drive the migration of these charge carriers across the interface. Specifically, electrons transfer from the CBM of Fe_2_O_3_ to MoS_2_, recombining with photogenerated holes in the VBM of MoS_2_ due to the influence of electric field I,^68^ which generates holes with strong oxidation properties in the VBM of Fe_2_O_3_. Similarly, photogenerated holes in the VBM of MoO_3_ migrate to Fe_2_O_3_, while electrons in the CBM of Fe_2_O_3_ move to MoO_3_ under the influence of electric field II. This results in the accumulation of photogenerated holes on Fe_2_O_3_, thereby promoting the water oxidation reaction.

**Figure 5:**
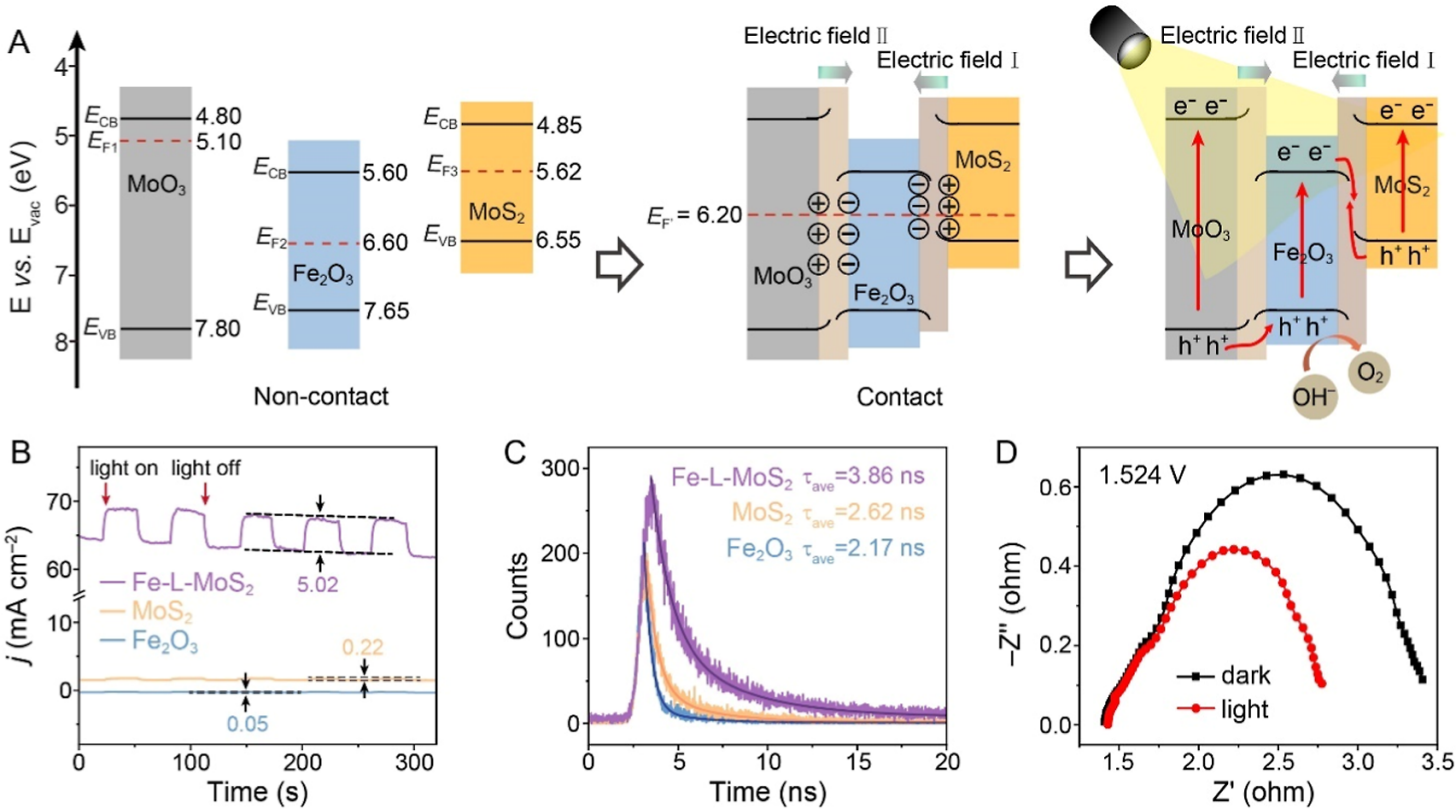
Mechanism of light-assisted OER. (A) Formation of built-in electric fields and migration routes of photogenerated carriers at the interfaces. *E*_CB_ and *E*_VB_ are conduction and valence band energies, respectively. (B) Transient photocurrent of Fe_2_O_3_, MoS_2_, and Fe-L-MoS_2_. (C) Time-resolved PL spectra of MoS_2_, Fe_2_O_3_, and Fe-L-MoS_2_. (D) Nyquist plots of Fe-L-MoS_2_ in the dark and under light irradiation (at 1.524 V vs RHE).

To investigate the photoresponse behavior, transient photocurrent was measured at a constant potential of 1.524 V. The current density of Fe-L-MoS_2_ increases immediately upon light exposure and returns to its initial level when the light is turned off, indicating a rapid photoresponse (Figure 5B). While MoS_2_ and Fe_2_O_3_ also exhibit photoresponse, their photocurrents (0.22 and 0.05 mA cm^–2^, respectively) are substantially lower than that of Fe-L-MoS_2_ (5.02 mA cm^–2^), further suggesting enhanced utilization efficiency of photogenerated carriers in the hybrid. Additionally, photoluminescence (PL) emission spectroscopy was employed to assess the recombination of photogenerated electron–hole pairs. A higher intensity in the PL spectrum typically indicates a greater recombination rate of photogenerated carriers.^69^ All catalysts exhibit a broad band in the range between 480 and 600 nm, suggesting multiple radiation processes of excited carriers (Figure S50). Notably, Fe-L-MoS_2_ displays lower emission intensity compared to MoS_2_ and Fe_2_O_3_, indicating that the three-phase heterointerfaces effectively mitigate carrier recombination. Time-resolved PL spectroscopy was further utilized to investigate charge dynamics. The carrier lifetime of Fe-L-MoS_2_ is determined to be 3.86 ns (Figure 5C and Table S7), which is 1.77 and 1.47 times longer compared to Fe_2_O_3_ (2.17 ns) and MoS_2_ (2.62 ns), respectively. This longer carrier lifetime can be ascribed to efficient carrier separation and reduced recombination. Moreover, the charge transfer resistance between the catalyst and electrolyte is decreased from 2.0 to 1.43 Ω under light irradiation (Figure 5D), reducing charge loss. This reduction is likely due to photogenerated holes acting as electron traps, attracting OH^–^ reactants.^34^ Consequently, the charge concentration of Fe-L-MoS_2_ increases nearly 5-fold under light irradiation (Figure S51), consistent with EIS and transient photocurrent results, further confirming that light irradiation generates more carriers to participate in catalytic reactions.

## Conclusions

This study demonstrates that the construction of photo- and electric-active MoO_3_/Fe_2_O_3_/MoS_2_ heterojunctions significantly enhances light-assisted seawater oxidation while simultaneously protecting Fe_2_O_3_ from chlorine-induced corrosion. The amorphous/crystalline interfaces in Fe-L-MoS_2_, characterized by a nanoparticle-decorated structure, expand the ECSA and provide abundant active sites. These heterointerfaces facilitate the OER process and, along with in situ leached MoO_4_^2–^ and SO_4_^2–^, mitigate Cl^–^ adsorption, thereby suppressing electrode corrosion. Additionally, built-in electric fields promote charge migration and decrease the recombination of photogenerated carriers, leading to increased participation of photocarriers in the OER process. As a result, Fe-L-MoS_2_ exhibits significantly enhanced OER activity and durability in alkaline seawater, achieving 10 mA cm^–2^ at 267 mV with remarkable long-term stability, showing only a 2.2% activity loss over 500 h at 300 mA cm^–2^. Light irradiation excites Fe-L-MoS_2_, generating carriers and reducing interfacial resistance, resulting in a 20.4% increase in seawater oxidation current density. When combined with a Pt/C cathode, the Fe-L-MoS_2_||Pt/C cell, assisted by light irradiation, requires only 2.476 V to reach 0.5 A cm^–2^ in seawater, demonstrating stable operation for over 170 h at 0.2 A cm^–2^. This study introduces a novel design concept for OER electrocatalysts that integrate excellent photoelectric synergistic effects with strong anticorrosion properties for scalable seawater electrolysis, offering a promising strategy for future energy applications.

## Supplementary Material



## Data Availability

Data is available upon reasonable request to the corresponding author.

## Notes

Chemicals, experimental methods, additional XRD, Raman, XPS, SEM, UPS, UV–vis spectrum, PL spectrum, cyclic voltammogram, electrochemical performance, DFT simulation results, and crystal structure (PDF)
