# Correction to “Corrosion-Resistant MoO_3_/Fe_2_O_3_/MoS_2_ Heterojunctions Stabilize OH^–^ Adsorption for Efficient Light-Assisted Seawater Electrooxidation”

**DOI:** 10.1021/jacs.5c19904

**Published:** 2025-12-06

**Authors:** Zhen Li, Wei Tao, Ying Wang, Xucun Ye, Yiqun Chen, Byungchan Han, Lawrence Yoon Suk Lee

The Acknowledgment section in the originally published article was corrected.

