# Supplementary material for: Corrosion-Resistant MoO3/Fe2O3/MoS2 Heterojunctions Stabilize OH– Adsorption for Efficient Light-Assisted Seawater Electrooxidation
Source: J Am Chem Soc. 2025 Jun 3;147(28):24461–72. doi: 10.1021/jacs.5c04085 (PMC12272696; doi:10.1021/jacs.5c04085)
Supplement: Supplementary file 1 [file ja5c04085_si_001.pdf]

Electronic Supplementary Information

Corrosion-Resistant $MoO_3/Fe_2O_3/MoS_2$ Heterojunctions Stabilize OH^- Adsorption for Efficient Light-Assisted Seawater Electrooxidation

Zhen Li,[1,‡] Wei Tao,[2,‡] Ying Wang,[1] Xucun Ye,[1] Yiqun Chen,[1] Byungchan Han[2,] and Lawrence Yoon Suk Lee[1,3,*]*

[1] Department of Applied Biology and Chemical Technology and Research Institute for Smart Energy, The Hong Kong Polytechnic University, Hung Hom, Kowloon, Hong Kong SAR, China

[2] Department of Chemical and Biomolecular Engineering, Yonsei University, Yonsei-ro 50, Seodaemun-gu, Seoul 03722, Republic of Korea

[3] Department of Chemical Engineering (Integrated Engineering), Kyung Hee University, 1732 Deogyeong-daero, Giheung-gu, Yongin-si, Gyeonggi-do 17104, Republic of Korea

[†] These authors contributed equally to this work.

*Corresponding Authors: bchan@yonsei.ac.kr (B. Han)
lawrence.ys.lee@polyu.edu.hk (L. Y. S. Lee)

1. Experimental Methods

Chemicals

Iron nitrate nonahydrate ($Fe(NO_3)_3 \cdot 9H_2O$, 99.9 %), molybdenum disulfide (MoS_2, 99 %), sodium sulfate (Na_2SO_4, 98 %), and potassium hydroxide (KOH, 99.99 %) were purchased from Sigma–Aldrich, USA. Sulfuric acid (H_2SO_4, 98.0 %), methanol (99.8 %), and acetone (99.8 %) were sourced from Duksan Chemicals, Korea. Ammonium molybdate tetrahydrate ($(NH_4)_6Mo_7O_{24} \cdot 4H_2O$, 83.0 %), sodium chloride (NaCl, 99.5 %), and potassium iodide (KI, 99.0 %) were obtained from Shenzhen Dieckmann Tech, China. Aqueous solutions were prepared using deionized (DI) water produced by a MilliQ Water System. Natural seawater (pH ~7.5) was collected from Tsim Sha Tsui near the Hong Kong Polytechnic University, Kowloon, Hong Kong SAR, China. All chemicals were used as received.

Synthesis of Fe-L-MoS_2

A pre-determined amount of $Fe(NO_3)_3 \cdot 9H_2O$ was added to acetone (5 mL) containing MoS_2 (20 mg) to form a uniform dispersion ($Fe(NO_3)_3$ concentration = 0.025, 0.05, and 0.1 M) by sonicating for 30 min. A Nd:YAG Q-switched pulsed laser (wavelength = 1,064 nm, Nimma-600 Laser system) with an energy output of 320 mJ (650 V) and energy stability (root mean square) $\leq$ 1 % was purchased from Beamtech Optronics Co. Ltd., China. The beam diameter was approximately 8 mm, and the MoS_2 suspension was laser-ablated under continuous stirring at 20 °C for pre-determined durations (5, 15, 25, and 35 min). Finally, the product was collected by centrifugation, thoroughly washed with DI water and ethanol, and dried at 60 °C under vacuum.

Synthesis of Fe_2O_3 and Fe_2O_3/MoS_2 Composites

For the synthesis of Fe_2O_3, $Fe(NO_3)_3 \cdot 9H_2O$ (1 mmol) was placed in a crucible and calcined at 200 °C for 0.5 h at a heating rate of 15 °C min^{-1} in an air atmosphere. Fe_2O_3/MoS_2 composites were synthesized using a conventional adsorption–calcination method. Specifically, MoS_2 (1 mmol) was dispersed in ethanol (20 mL) by sonicating for 30 min. To this suspension, $Fe(NO_3)_3 \cdot 9H_2O$ (2 mmol) was added and sonicated for 30 min. The mixture was then steam dried at 90 °C with continuing stirring, followed by calcination at 200 °C for 0.5 h at a heating rate of 15 °C min^{-1} in an air atmosphere.[1]

Fabrication of Pt/C and RuO_2 Electrodes

Catalyst ink was prepared by dissolving 20 wt.% Pt/C or RuO_2 (5 mg) in a mixed solution of ethanol (960 μL) and 5 wt.% Nafion (40 μL), followed by sonication for 60 min. Subsequently, catalyst ink (50 μL) was drop-cast onto pre-treated nickel foam (0.25 cm^2).

Materials Characterization

The phase and crystallinity of the as-synthesized samples were determined using XRD (Rigaku SmartLab) with Cu K_{α} radiation at a scanning rate of 10° min^{-1} over a 2θ range from 10° to 80°. Raman spectra were recorded on a confocal micro-Raman spectroscopy system (Renishaw, inVia) with a 785 nm streamline laser excitation. Morphology and size distribution were analyzed by a field-emission SEM (Tescan MIRA). Lattice fringes and elemental distribution were examined using a scanning TEM (JEM-2100F STEM) equipped with an EDS. Chemical bonding states and elemental composition were investigated by an XPS (ESCALAB 250 Xi, Thermo Fisher) equipped with monochromatic Al K_{α} radiation. Elemental compositions were determined using ICP-OES on an Agilent 710 Series spectrometer. BET adsorption–desorption isotherms and specific surface area measurements were conducted through physical adsorption of N_2 at −195.85 °C using a MicroActive ASAP 2460 apparatus. UV–Vis spectra were collected on a Jasco V780 spectrophotometer. UPS was performed on a ThermoFisher EscaLab 250 Xi spectrometer to determine work functions. PL spectra were measured on an FLS1000 fluorescence spectrophotometer with a 375 nm excitation wavelength. Time-resolved PL decay curves were recorded with a 375 nm pulsed laser on the same spectrophotometer. The obtained decay curves were fitted using a bi-exponential kinetic function:

$$\tau_{ave} = A_1 \exp\left(-\frac{t}{\tau_1}\right) + A_2 \exp\left(-\frac{t}{\tau_2}\right) \quad (1)$$

where A_1 and A_2 are the corresponding amplitudes and τ_1 and τ_2 are the emission lifetimes.

Light-Assisted Electrocatalysis Measurements

A Xe lamp (PLS-SXE300+, Beijing Perfectlight) equipped with a UV–Vis filter was used as the light source (350 nm ≤ λ ≤ 780 nm) and positioned at a constant distance of 26 cm from one side of the electrolytic cell. A circulating cooling water system maintained the electrolyte temperature at 23 °C, with a light intensity of approximately 200 mW cm^{-2} on the electrode surface. The OER catalysis in seawater was conducted under the same conditions with light irradiation. For seawater splitting experiments, a Pt/C cathode (0.25 cm^2) was coupled with a Fe-L-MoS_2 anode (0.25 cm^2) to form a full-cell water-splitting system (**Figure S1**). For comparison, commercial RuO_2 and Pt/C on Ni foam were used as benchmarks for the anode and cathode, respectively.

Electrochemical Measurements

Electrochemical tests were conducted in a standard three-electrode configuration in 1.0 M KOH electrolyte, with a graphite rod and a Hg/HgO electrode serving as the counter and reference electrodes, respectively. All data were collected using an electrochemical station (CHI760E). For

the OER experiments, a homogeneous catalyst ink (5 mg mL^{-1}) was first prepared by dissolving the catalyst in ethanol (960 μL), along with Nafion (40 μL) and carbon conductor (VXC-72, 2 mg). Nickel foam was cleaned by sonication in acetone and washed with 3 M HCl for 10 min to remove the surface oxide layer. Catalyst ink (25 μL) was then pipetted onto a nickel foam (0.25 cm^2) and allowed to dry naturally, serving as the working electrode. All potentials in this work are reported against the reversible hydrogen electrode (RHE, $E_{RHE} = E_{Hg/HgO} + 0.059 \times pH + 0.098$). Before testing, the working electrode was stabilized by performing 100 cyclic voltammogram (CV) cycles within a potential range between 1.124 and 1.624 V. LSV was conducted from 1.2 to 1.8 V at a scan rate of 5 mV s^{-1}. All polarization curves were corrected for ohmic losses with 85 % *i*R compensation. All measurements were repeated at least three times. EIS was performed at 1.524 V from 100 kHz to 0.1 Hz, with an amplitude of 10 mV.

Mott–Schottky plots were obtained at a frequency of 800 Hz using a saturated calomel electrode (SCE) and a Pt wire as the reference and counter electrode, respectively. The charge concentration was evaluated from the Mott–Schottky plots obtained in Na_2SO_4 (0.2 M) at 800 Hz. Carrier density (N_D) was calculated using the following equation.

$$N_D = -\frac{2}{e\varepsilon_0\varepsilon_r}\frac{dV}{d\frac{1}{C^2}} \quad (2)$$

where e, C, V, ε_0, and ε_r are elementary charge (1.6×10^{-19} C), specific capacity, applied voltage, vacuum permittivity (8.85×10^{-14} F cm^{-1}), and dielectric constant, respectively.

Electrochemical surface area (ECSA) was estimated by measuring double-layer capacitances (C_{dl}) from CVs collected in a non-Faradaic region between 1.125 and 1.225 V (*vs*. RHE) at various scan rates of 20, 40, 60, 80, and 100 mV s^{-1} using the equation:[1]

$$ECSA = S_{GA} \times \frac{C_{dl}}{C_s} \quad (3)$$

where S_{GA} is the geometric area of the glassy carbon electrode (GCE, 0.196 cm^2), C_s is the specific capacitance of the sample or the capacitance of an atomically smooth planar surface of the material per unit area under identical electrolyte conditions. For our surface area estimates, we used a general specific capacitance of $C_s = 0.04$ mF cm^{-2} based on the typical reported value.[2, 3]

Turnover frequency (TOF) values were calculated using the equation:

$$TOF = \frac{i}{nFN_{site}} \quad (4)$$

where i is the current at a given OER overpotential, F is the Faradaic constant (96,485 C mol^{-1}), n is the number of electrons transferred to produce one O_2 molecule (4 for OER), and N_{site} is the total number of metal sites (mole) on the electrode, determined by ICP-OES.

Apparent electrochemical activation enthalpy (E_a) for OER was calculated from the slope of the Arrhenius plot:

$$\frac{\partial(ln i_0)}{\partial(\frac{1}{T})} = \frac{-E_a}{R} \quad (5)$$

where R is the universal gas constant, T is the absolute temperature, and i_0 is the kinetic current density.

To determine Faradaic efficiency (FE), the gaseous product generated at the anode was measured using a drainage method in a gas-tight H-cell at a fixed current density of 100 mA cm^{-2} in 1 M KOH seawater electrolyte. The O_2 product was collected in a graduated tube with one end sealed. The FE was calculated using the total charge passed through the electrode (Q) and the amount of produced oxygen (n) according to the following equation:

$$\mathrm{FE} = \frac{4F \times n}{Q} \quad (6)$$

Hypochlorite titration analysis was conducted to identify the formation of hypochlorite (ClO^-). Immediately after the FE test, iodide titration was conducted. Hypochlorite reacts with I^-, leading to an instantaneous color change and a distinct absorption peak at around 358 nm. Briefly, after the FE test, 10 mL of electrolyte was pipetted from the reaction cell. The pH of the extracted electrolyte was adjusted to 5 using 0.5 M H_2SO_4, followed by the addition of 0.5 M KI solution (5 mL). The solution was then titrated dropwise with 0.01 M thiosulfate solution. Subsequently, a UV−vis spectrum was collected using a spectrophotometer (Agilent Cary-60) within the wavelength range of 300 to 600 nm. The presence of hypochlorite was confirmed by monitoring the peak intensity at 358 nm.

In situ Raman spectra were recorded on a Raman spectrometer equipped with a 785 nm streamlined laser equipped with a working station (CHI760E). Each spectrum was accumulated twice to enhance signal quality with 10 s exposure time and 0.5 % laser intensity (the power was reduced to 1.5 mW). A standard three-electrode configuration was used with a Hg/HgO reference electrode and a Pt wire counter electrode for these measurements. LSV scans were conducted at a scan rate of 0.25 mV s^{-1} to allow for sufficient time for Raman spectra acquisition.

Quasi-*in-situ* UV−vis spectra were collected using a spectrophotometer (Jasco V780) and a potentiostat (CHI760E) to identify the presence of MoO_4^{2-} and SO_4^{2-} during the reconstruction of Fe-L-MoS_2. Using ammonium molybdate ($(NH_4)_6Mo_7O_{24}\cdot 4H_2O$) and Na_2SO_4 as references

for MoO_4^{2-} and SO_4^{2-} detection, respectively, standard absorption spectra were first obtained for solutions containing 0.1 mM ($(NH_4)_6Mo_7O_{24}\cdot 4H_2O$) and 0.01 mM Na_2SO_4. The characteristic absorption peaks for MoO_4^{2-} were identified at 211 and 232 nm, and the SO_4^{2-} peak at 216 nm. To detect MoO_4^{2-} and SO_4^{2-} during the OER process, UV−vis absorption spectra of the electrolytes were collected at different reaction times. A two-electrode configuration was used, with Fe-L-MoS_2 as the anode and a Pt wire as the cathode, using 3 mL of the reaction reagent as the electrolyte. An applied potential of 2.5 V was maintained using chronoamperometry for 1 min, and the entire electrolyte was extracted from the cell and analyzed using UV–vis spectroscopy. The electrolyte (3 mL) was added to the cell for subsequent measurements.

Photo-to-current efficiency (PCE) was determined using the equation:

$$\text{PCE} = \frac{n_{O_2} \times \Delta G_0}{P_{\text{total}} \times t} \times 100\ \% = \frac{\frac{I \times t}{4e \times 6.02 \times 10^{23}} \times 4.74 \times 10^5 \times \text{FE}}{P_{\text{total}} \times t} \times 100\ \% \qquad (7)$$

where n_{O2} is the moles of O_2 produced, ΔG_0 is the Gibbs free energy change per mole of O_2 (4.74×10^5 J mol^{-1}), I is the photocurrent, t is the working time, FE is the Faradaic efficiency, and P_{total} is the optical power (0.05 W).

Theoretical Simulations

Density functional theory (DFT) +U calculations were conducted using the plane wave basis set implemented in the Vienna *Ab initio* Simulation Package.[4-6] Projected augmented-wave (PAW) potentials and Perdew−Burke−Enzerhof (PBE) functional within a generalized gradient approximation (GGA) framework were employed to accurately represent electron–ion and exchange-correlation interactions.[7-9] An energy cutoff of 530 eV and a stringent force convergence criteria of 0.02 eV·Å^{-1} were applied to ensure precision. Grimme's DFT-D3 method was applied to account for Van der Waals interactions.[10] The Hubbard correction (U) was further introduced in the system with a U-parameter of 5.3 for Fe.[11] The MoO_3/Fe_2O_3 amorphous structure was generated using *ab initio* molecular dynamics (AIMD),[12] and subsequently combined with the MoS_2 substrate to construct the amorphous/crystalline $MoO_3/Fe_2O_3/MoS_2$ heterostructure. The Monkhorst-Pack scheme with a 3 × 3 × 1 k-mesh was employed for Brillouin zone sampling. Bader charge analysis was conducted to determine the partial charges of individual atoms within the materials.[13] The charge density difference (Δρ) was calculated using the following equation:

$$\Delta\rho = \rho_{total} - \rho_{MoS_2} - \rho_{MoO_3/Fe_2O_3} \qquad (8)$$

The adsorption energy was calculated using:

$$E_{ads} = E_{X/slab} - E_X - E_{slab} \qquad (9)$$

where $E_{X/slab}$, $E_{X,}$ and E_{slab} are the total energy of the slab with the adsorbate, energy of the free adsorbate (X), and energy of the slab, respectively. The Gibbs free energy (ΔG) was calculated using equation (9):

$$\Delta G = \Delta E + \Delta ZPE - T\Delta S + \Delta G_U + \Delta G_{pH} \quad (10)$$

where ΔE is reaction energy change, ΔZPE is zero-point energy, T is temperature (set to 298.15 K in this study), and ΔS is entropy change. Electrode potential bias (ΔG_U) was calculated as $\Delta G = -neU$, where U refers to the applied potential (*vs*. RHE), n is the number of proton–electron pairs transferred, and e is the elementary charge. ΔG_{pH} is the correction for pH influence, which was defined as $\Delta G_{pH} = -k_B T \ln 10 \cdot Ph$, where k_B is the Boltzmann constant, and pH is 14 for alkaline solution.

The reaction mechanism and free energy calculation are shown in the following:

Oxygen Evolution Reaction (OER). The OER occurs in an alkaline solution through four concerted proton–electron transfer (CPET) reactions as follows:

$$* + OH^- \rightarrow *OH + e^- \quad (11)$$

$$*OH + OH^- \rightarrow *O + H_2O\ (l) + e^- \quad (12)$$

$$*O + OH^- \rightarrow *OOH + e^- \quad (13)$$

$$*OOH + OH^- \rightarrow * + O_2\ (g) + H_2O\ (l) + e^- \quad (14)$$

where * represents the adsorption site. The adsorption energies of the intermediates were calculated using the following equations:

$$\Delta G_{*OH} = E_{*OH} + \Delta ZPE - T\Delta S - E_* - (G_{H_2O} - 0.5G_{H_2}) \quad (15)$$

$$\Delta G_{*O} = E_{*O} + \Delta ZPE - T\Delta S - E_* - (G_{H_2O} - G_{H_2}) \quad (16)$$

$$\Delta G_{*OOH} = E_{*OOH} + \Delta ZPE - T\Delta S - E_* - (2G_{H_2O} - 1.5G_{H_2}) \quad (17)$$

As such, the Gibbs free energy changes for each step can be calculated using the following equations:

$$\Delta G_1 = \Delta G_{*OH} - eU + \Delta G_{pH} \quad (18)$$

$$\Delta G_2 = \Delta G_{*O} - \Delta G_{*OH} - eU + \Delta G_{pH} \quad (19)$$

$$\Delta G_3 = \Delta G_{*OOH} - \Delta G_{*O} - eU + \Delta G_{pH} \quad (20)$$

$$\Delta G_4 = 4.92 - \Delta G_{*OOH} - eU + \Delta G_{pH} \quad (21)$$

The overpotential for OER was determined using following equation:

$$\eta_{OER} = \frac{\{max[\Delta G_n]\}}{e} - 1.23\ V \quad (22)$$

where ΔG_n are the free energies of each elementary steps (n = 1 – 4).

Chlorine Evolution Reaction (CER). The CER was analyzed using a two-electron process *via* the Volmer–Heyrovsky mechanism. The CER activity was evaluated by the change in Gibbs free energy associated with chlorine adsorption (ΔG_{*Cl}):

$$* + Cl^{-} \rightarrow *Cl + e^{-} \quad (23)$$

$$\Delta G_{*Cl} = \Delta E_{*Cl} + \Delta ZPE - T\Delta S - eU + 1.36 \quad (24)$$

The overpotential for CER was determined using following equation:

$$\eta_{CER} = \frac{|\Delta G_{*Cl}|}{e} \quad (25)$$

2. Supplementary Figures

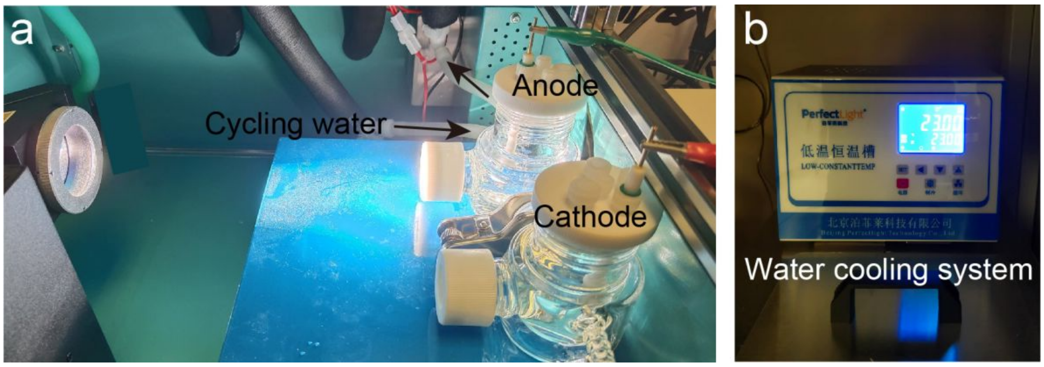

Figure S1. (a) Photo of an H-type electrolyzer equipped with a lamp and (b) cooling system for the control of the electrolyte's temperature.

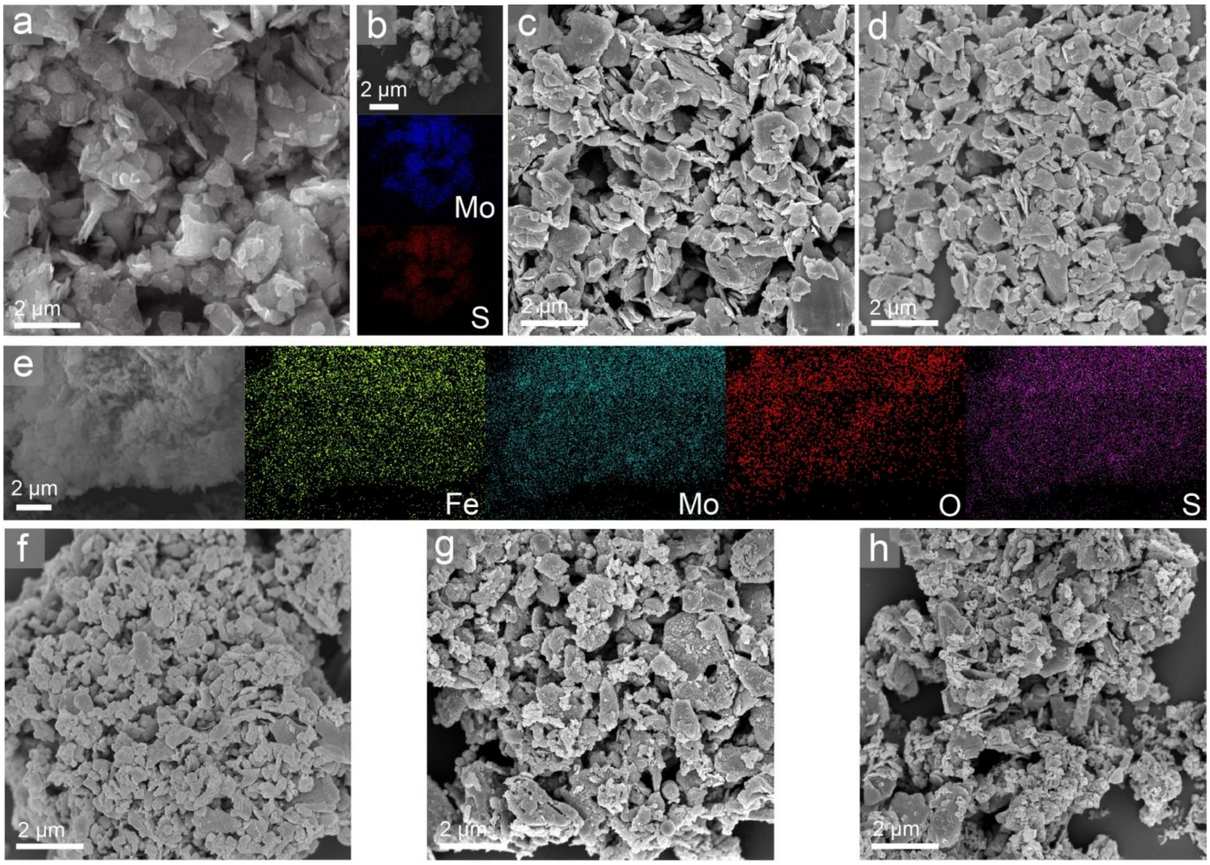

Figure S2. SEM images of (a) bulk MoS_2 and MoS_2 samples laser-ablated for various durations with $[Fe(NO_3)_3]$ = 0.5 M: (c) 5 min, (d) 15 min, and (f) 35 min and with various $Fe(NO_3)_3$ concentrations for 25-min laser ablation: (g) 0.025 M and (h) 0.1 M $Fe(NO_3)_3$. The corresponding EDS mapping images for (b) bulk MoS_2 and (e) Fe-L-MoS_2 (25 min, 0.5 M $Fe(NO_3)_3$.

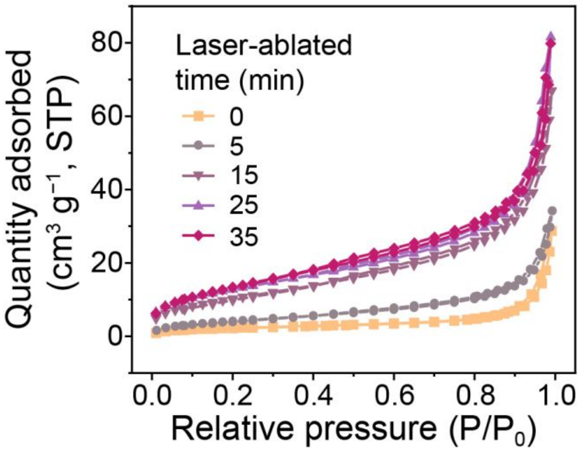

Figure S3. Adsorption–desorption isotherms of pristine MoS_2 and L-Fe-MoS_2 samples synthesized using different laser-ablation times.

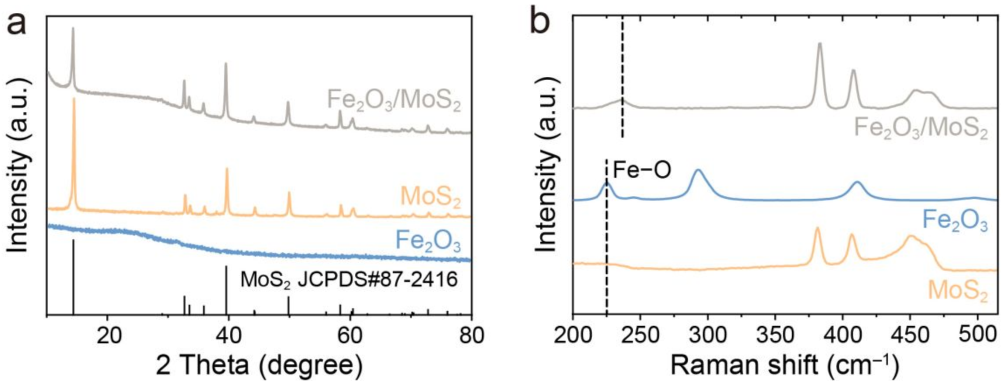

Figure S4. (a) XRD patterns and (b) Raman spectra of MoS_2, Fe_2O_3, and Fe_2O_3/MoS_2.

No obvious diffraction peak is observed from Fe_2O_3 (**Figure S4a**), consistent with previous reports,[1] suggesting its amorphous structure. The XRD pattern of Fe_2O_3/MoS_2 heterostructure displays strong peaks corresponding to MoS_2 with a minor Fe−O band, indicating the existence of amorphous Fe_2O_3 in the hybrid. However, this Fe−O band is red-shifted compared to pristine Fe_2O_3 (**Figure S4b**), which can be ascribed to the random strain effect resulting from the surface functionalization of MoS_2.[14, 15]

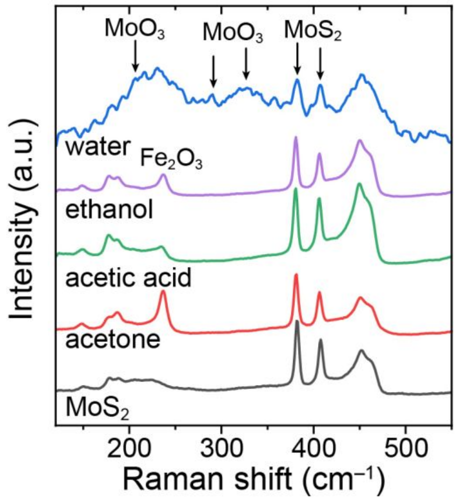

Figure S5. Raman spectra of samples synthesized in water, ethanol, acetic acid, and acetone.

Raman spectra reveal that Fe-L-MoS_2 synthesized in organic solvents (ethanol, acetic acid, and acetone) exhibits a significantly reduced MoO_3 peak compared to that prepared in water, indicating that water promotes MoS_2 oxidation due to free oxygen generated from water dissociation. In contrast, organic solvents produce reducing gases such as CH_4 and CO,[16] which suppress excessive MoS_2 oxidation. Additionally, Raman peaks at 237 cm^{-1} (Fe_2O_3) and 381 cm^{-1} (MoS_2) show a peak intensity ratio (I_{237}/I_{381}) of 0.72 for acetone, 0.36 for ethanol, and 0.21 for acetic acid, reflecting greater Fe_2O_3 formation in acetone. The lower polarity of acetone, compared to ethanol and acetic acid, weakens its interactions with Fe^{3+} ions, enhancing Fe^{3+} adsorption on MoS_2 and thus promoting Fe_2O_3 deposition. Consequently, acetone optimizes Fe_2O_3 formation while mitigating MoS_2 oxidation.

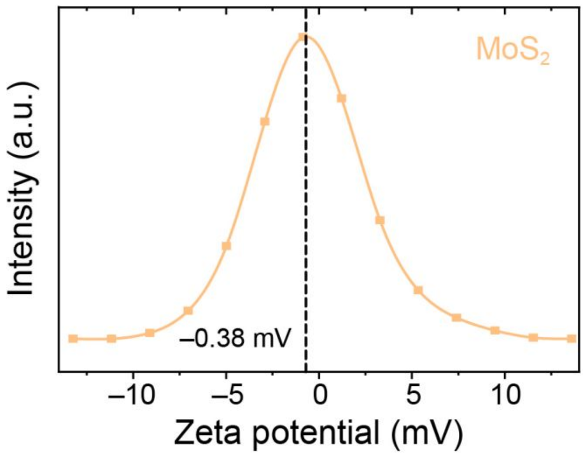

Figure S6. Zeta potential of pristine MoS_2.

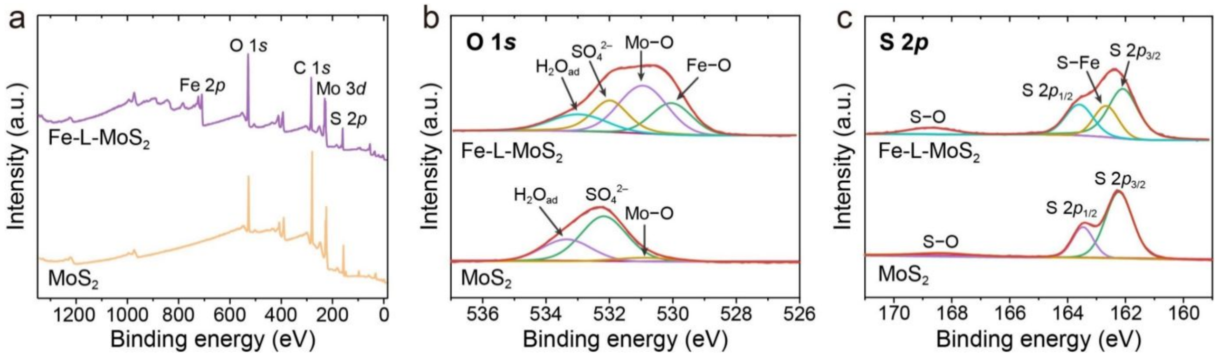

Figure S7. (a) XPS survey, (b) O 1*s*, and (c) S 2*p* spectra of pristine MoS_2 and Fe-L-MoS_2.

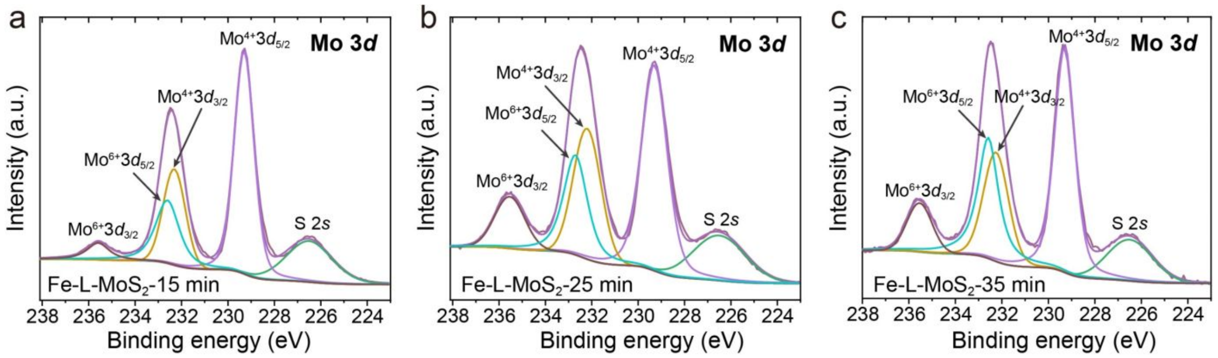

Figure S8. XPS Mo 3*d* spectra of Fe-L-MoS_2 prepared using various laser-ablation times: (a) 15 min, (b) 25 min, and (c) 35 min.

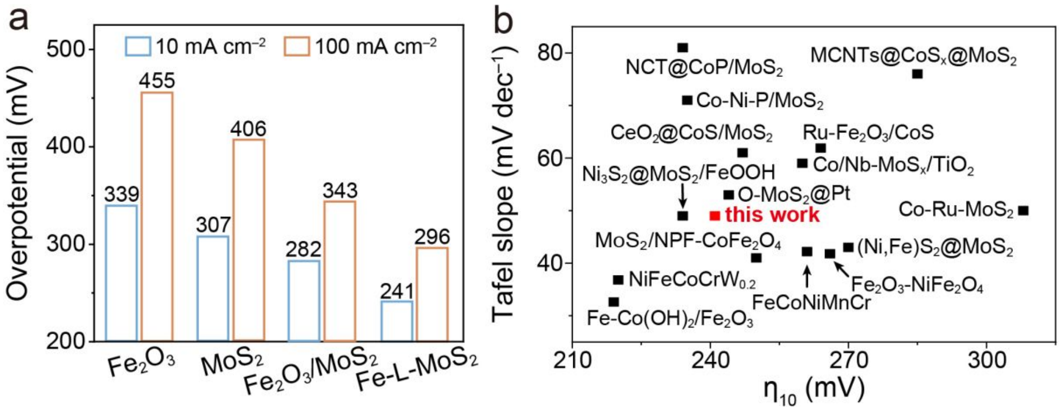

Figure S9. (a) Comparison of OER overpotentials required for MoS_2, Fe_2O_3, Fe_2O_3/MoS_2, and Fe-L-MoS_2 to achieve 10 and 100 mA cm^{-2}. (b) Comparison of OER overpotentials and Tafel slopes of Fe-L-MoS_2 with previously reported OER catalysts.

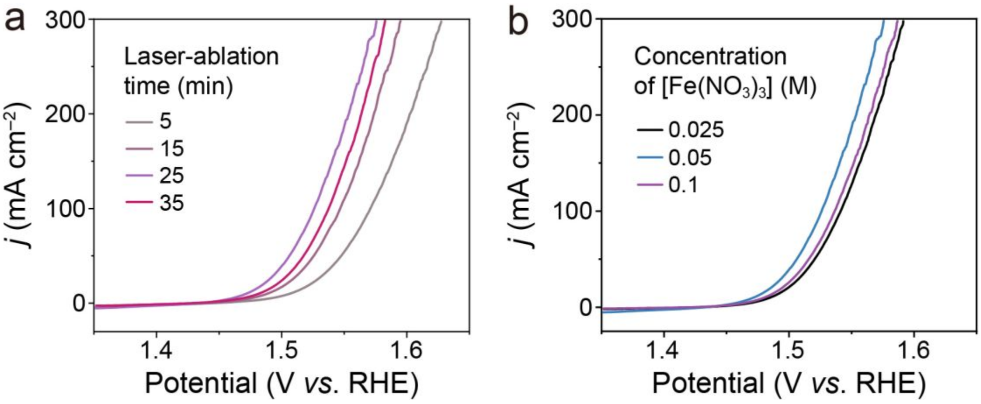

Figure S10. LSV curves of Fe-L-MoS_2 samples prepared using various (a) laser-ablation times and (b) $Fe(NO_3)_3$ concentrations.

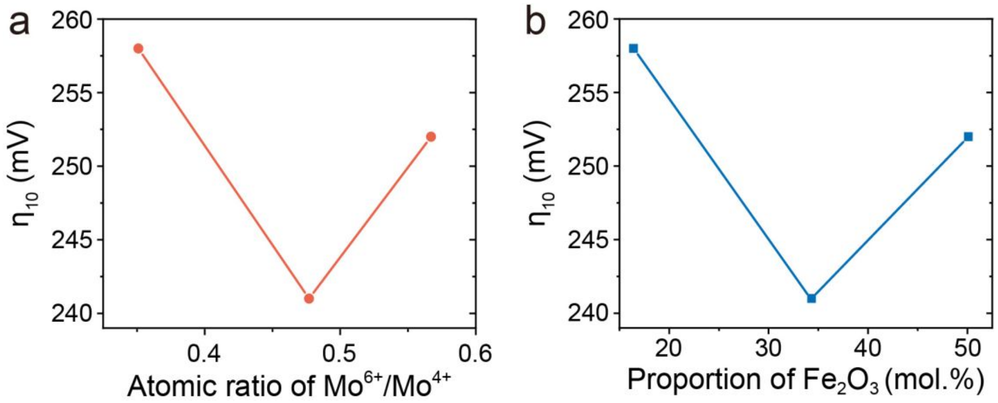

Figure S11. Correlation of OER overpotential at 10 mA cm^{-2} (η_{10}) with (a) Mo^{6+}/Mo^{4+} ratio and (b) Fe_2O_3 content.

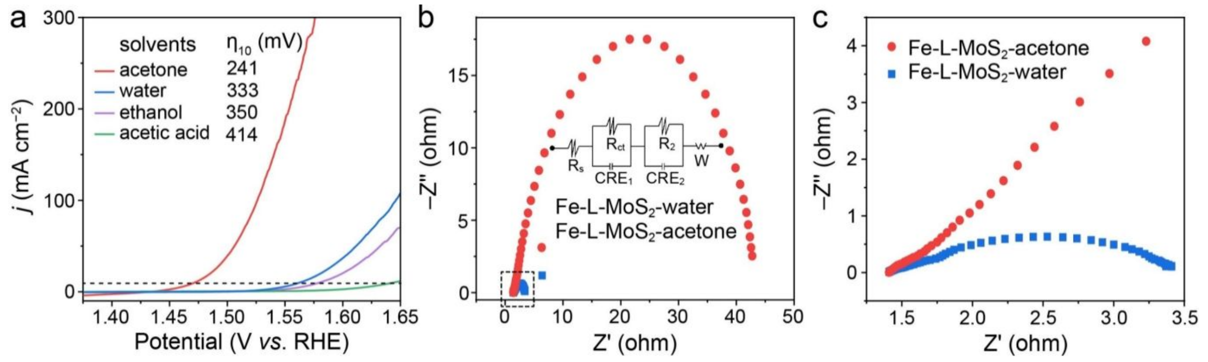

Figure S12. (a) LSVs of Fe-L-MoS_2 synthesized in various solvents. (b) Nyquist plots of Fe-L-MoS_2-acetone and Fe-L-MoS_2-water, with the inset showing the equivalent circuit (R_s: electrolyte resistance; CPE_1: double-layer capacitance; R_{ct}: interfacial charge transfer reaction, W: Warburg impedance; and CPE_2 and R_2: dielectric properties and electrode resistance) (c) Enlarged view of the dotted region in (b).

Fe-L-MoS_2 synthesized in acetone exhibits superior OER activity compared to samples prepared in water, ethanol, or acetic acid (**Figure S12a**). The product synthesized in water shows higher electron transfer resistance (**Figures S12b** and **S12c**), attributed to excessive MoS_2 oxidation.

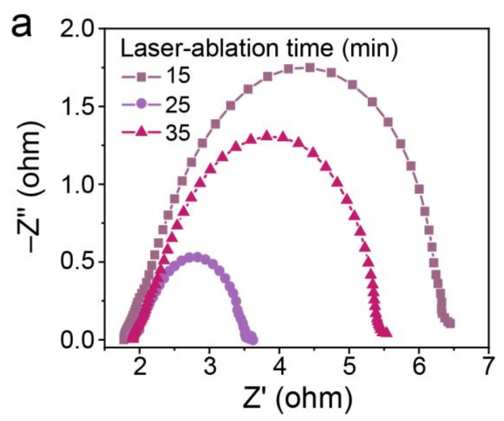

Figure S13. Nyquist plots of products synthesized using various (a) laser-ablation times and (b) $Fe(NO_3)_3$ concentrations.

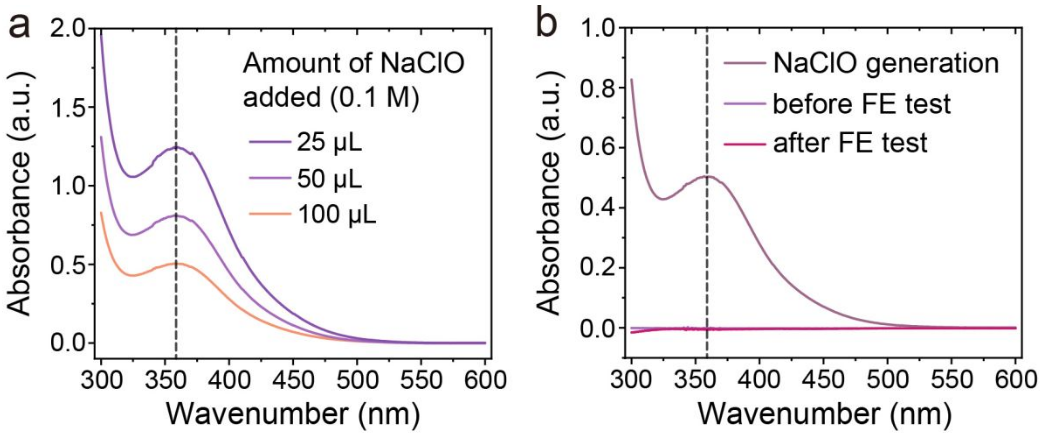

Figure S14. UV−Vis spectra of (a) iodide titration with various NaClO concentrations and (b) electrolytes before and after the FE test.

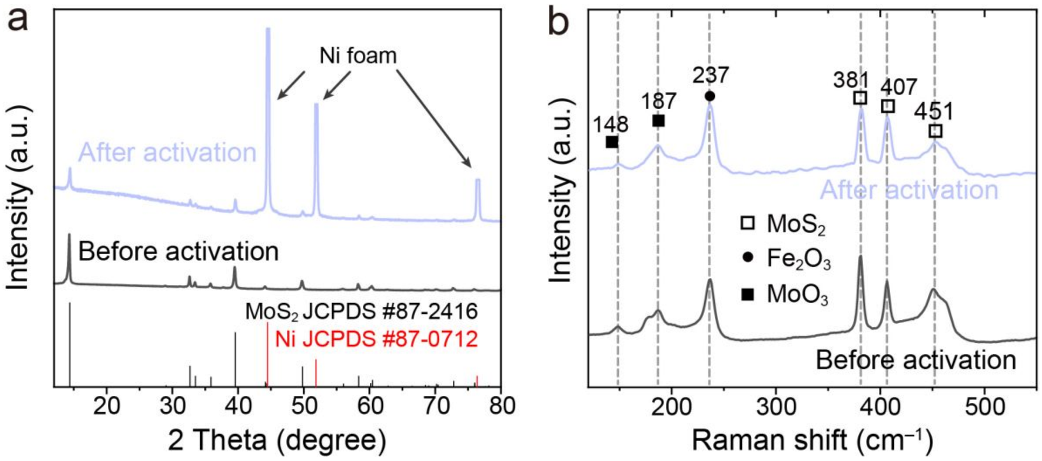

Figure S15. (a) XRD patterns and (b) Raman spectra of Fe-L-MoS_2 before and after OER activation.

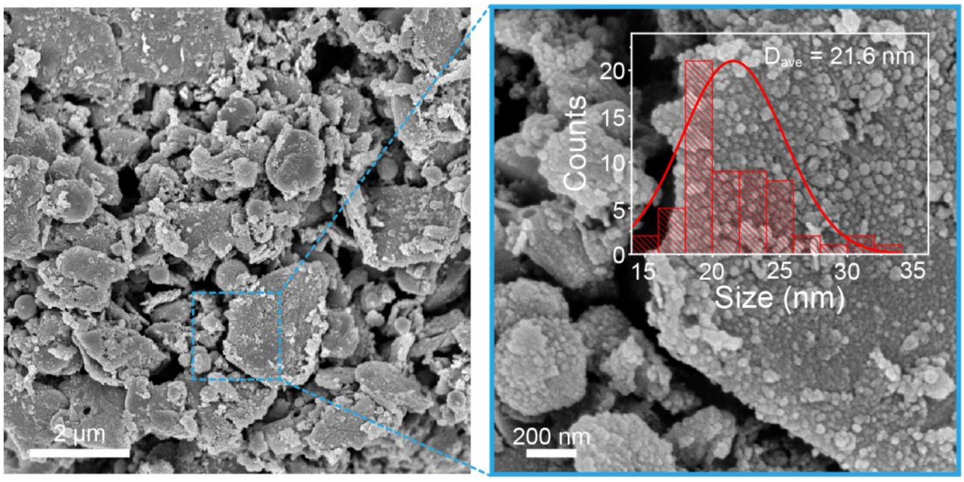

Figure S16. SEM images of Fe-L-MoS_2 after OER activation.

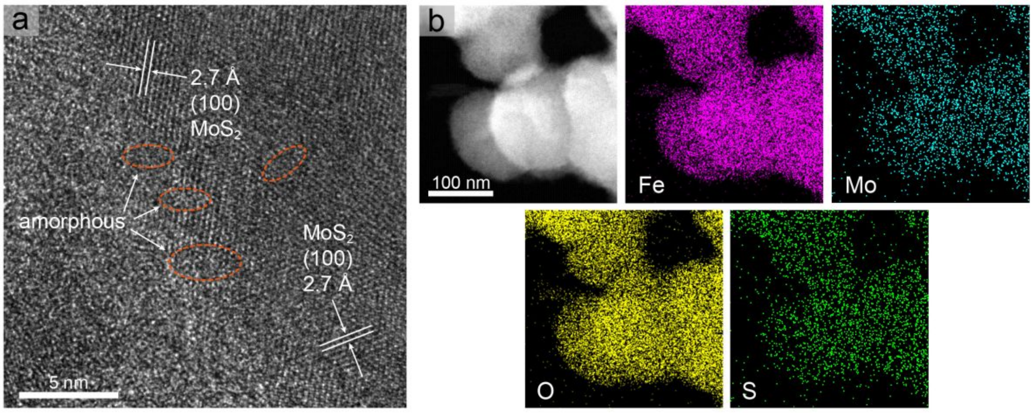

Figure S17. (a) High-resolution TEM and (b) STEM images with the corresponding EDS mapping images of Fe-L-MoS_2 after OER activation.

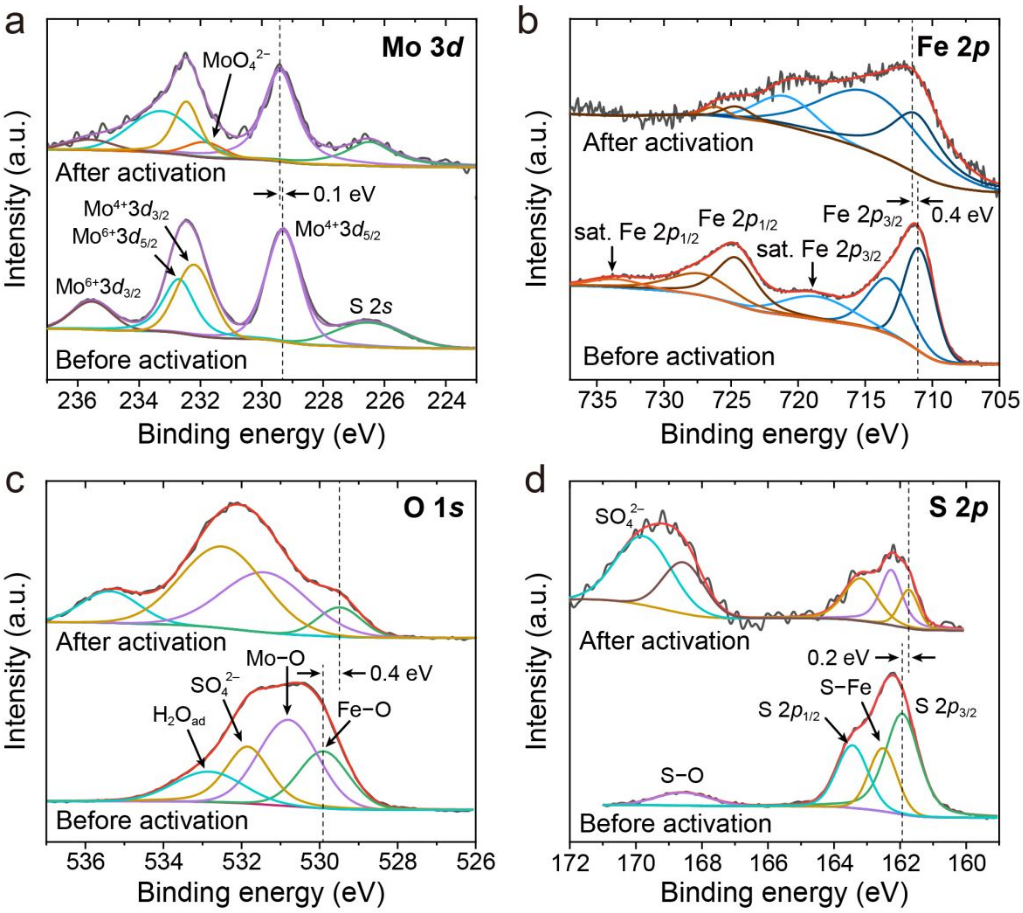

Figure S18. XPS (a) Mo 3*d*, (b) Fe 2*p*, (c) O 1*s*, and (d) S 2*p* spectra of Fe-L-MoS_2 before and after OER activation.

Figure S18a presents the XPS analysis of Fe-L-MoS_2 in the Mo 3*d* region before and after OER activation. A slight shift of 0.1 eV towards higher binding energy is observed on Mo $3d_{5/2}$ after OER activation, indicating an increase in the valence state of Mo. The Mo^{6+}/Mo^{4+} ratio rises from 0.48 to 0.53, suggesting a reduction in Mo^{4+} species. Additionally, a new peak at 231.85 eV is attributed to MoO_4^{2-}.[17] Similarly, the Fe 2*p* spectrum exhibits a 0.4-eV shift towards higher binding energy (**Figure S18b**), indicating an increased valence state of Fe following OER activation. In contrast, redshifts of 0.4 and 0.2 eV are observed in the XPS O 1*s* and S 2*p* regions (**Figure S18c** and **S18d**), reflecting an increased electronic density around O and S atoms. The emergence of a new peak at 169.8 eV is attributed to SO_4^{2-}.[18]

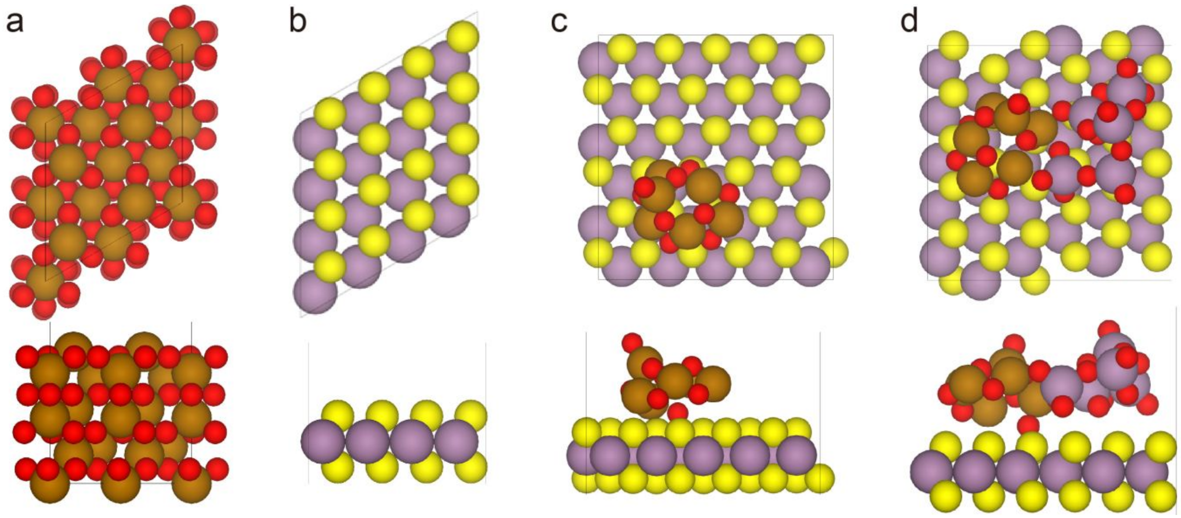

Figure S19. Optimized atomic structures of (a) Fe_2O_3, (b) MoS_2, (c) Fe_2O_3/MoS_2, and (d) $MoO_3/Fe_2O_3/MoS_2$. Mo, Fe, O, and S atoms are shown in violet, brown, red, and yellow, respectively.

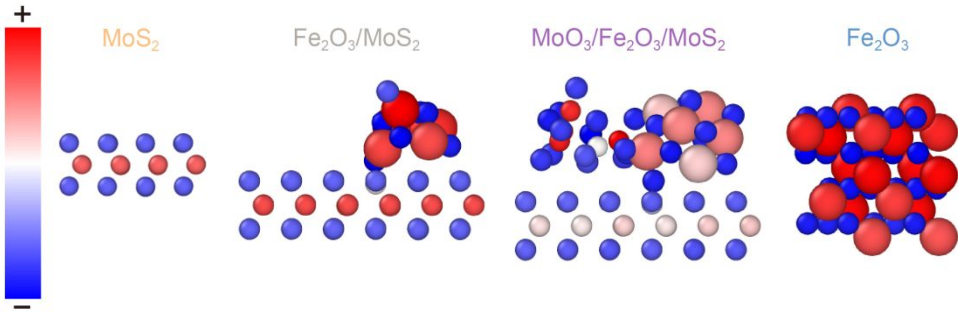

Figure S20. Bader charge distributions in Fe_2O_3, MoS_2, Fe_2O_3/MoS_2, and $MoO_3/Fe_2O_3/MoS_2$ heterointerface.

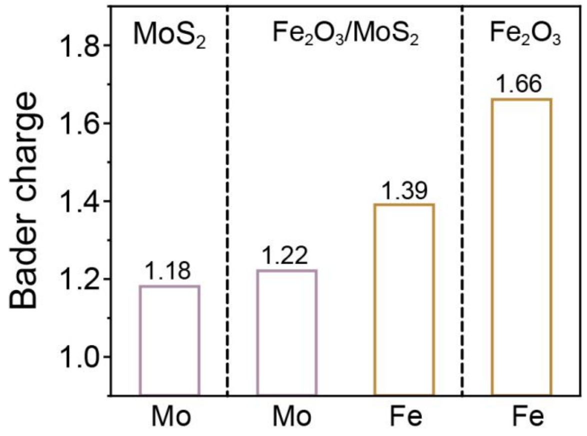

Figure S21. Bader charges at Mo and Fe sites of Fe_2O_3, MoS_2, and Fe_2O_3/MoS_2 heterointerface.

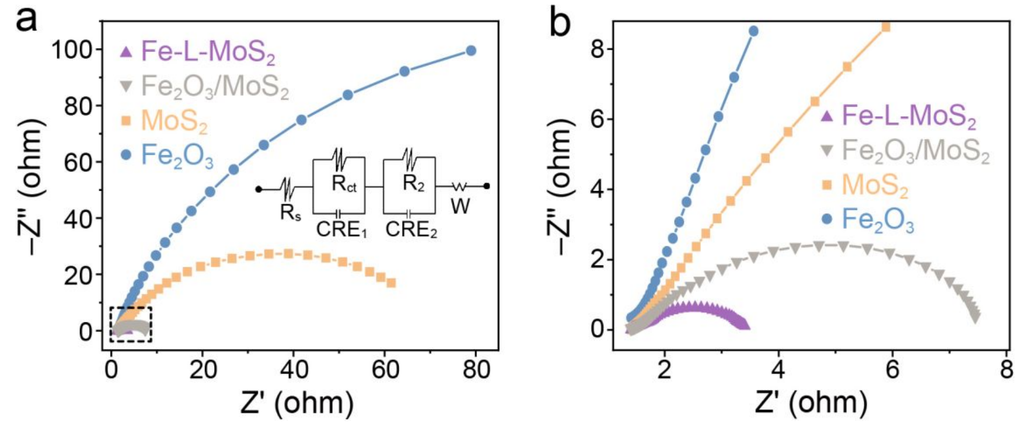

Figure S22. (a) Nyquist plots of MoS_2, Fe_2O_3, Fe_2O_3/MoS_2, and Fe-L-MoS_2. **(b)** Enlarged region of dotted box in (a). Inset is an equivalent circuit, where R_s stands for the electrolyte resistance, CPE_1 represents double-layer capacitance, R_{ct} is related to the interfacial charge transfer reaction, W is Warburg, and CPE_2 and R_2 are associated with the dielectric properties and the resistance of the electrode itself.

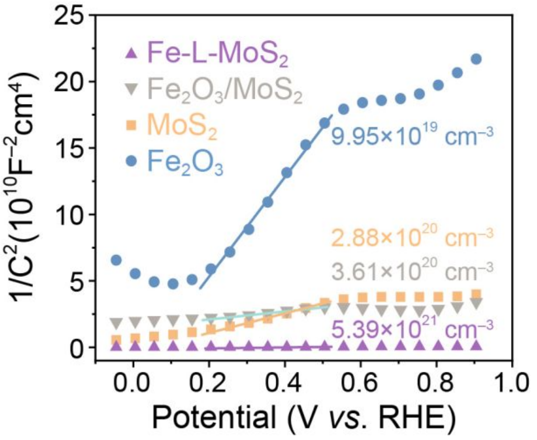

Figure S23. Mott–Schottky plots of MoS_2, Fe_2O_3, Fe_2O_3/MoS_2, and Fe-L-MoS_2.

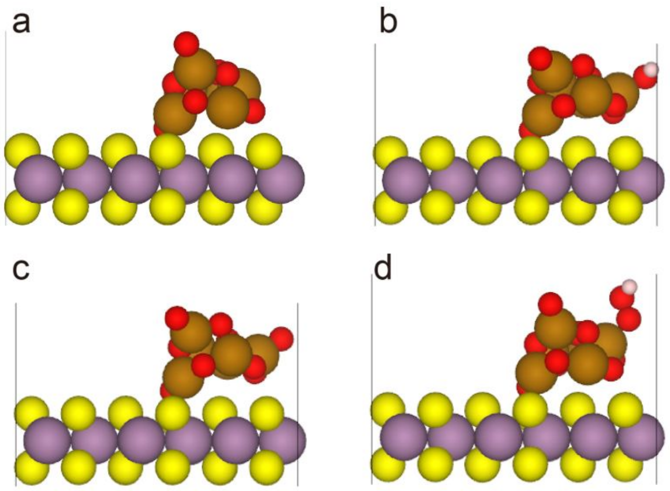

Figure S24. Structures of (a) Fe_2O_3/MoS_2 heterojunction, and (b) *OH-, (**c**) *O-, and (**d**) *OOH-adsorbed Fe_2O_3/MoS_2 (on Fe sites). Mo, Fe, O, S, and H atoms are shown in violet, brown, red, yellow, and pink, respectively.

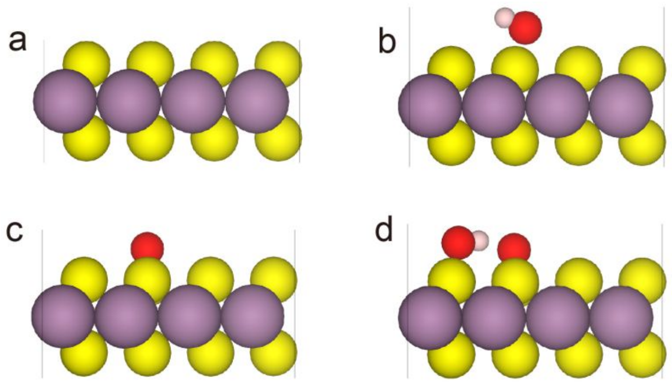

Figure S25. Structures of (a) MoS_2, (b) *OH-, (c) *O-, and (d) *OOH-adsorbed MoS_2 (on S sites). Mo, O, S, and H atoms are shown in violet, red, yellow, and pink, respectively.

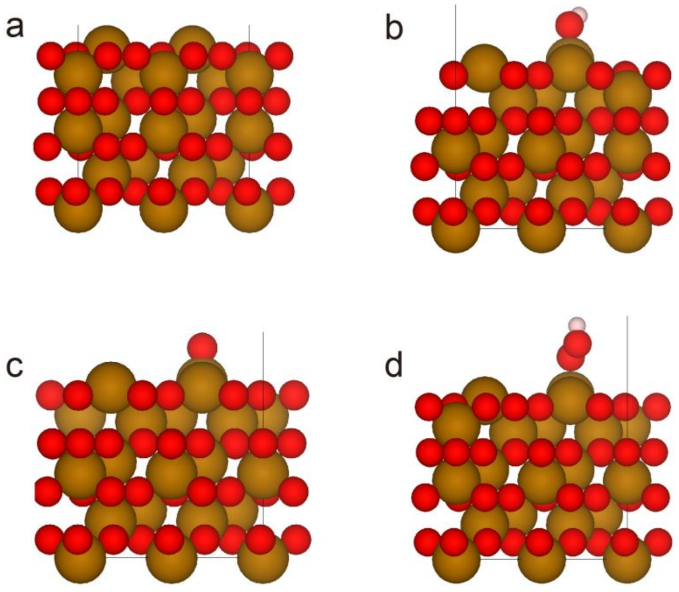

Figure S26. Structures of (a) Fe_2O_3, (b) *OH-, (c) *O-, and (d) *OOH-adsorbed MoS_2 (on S sites). Fe, O, and H atoms are shown in brown, red, and pink, respectively.

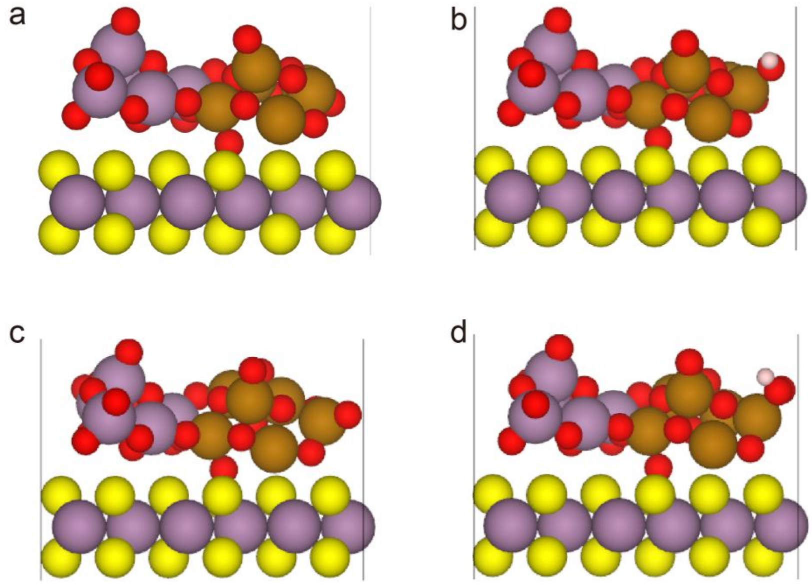

Figure S27. Structures of (a) $MoO_3/Fe_2O_3/MoS_2$ heterojunction, and (b) *OH-, (c) *O-, and (d) *OOH-adsorbed $MoO_3/Fe_2O_3/MoS_2$ (on Fe sites). Mo, Fe, O, S, and H atoms are shown in violet, brown, red, yellow, and pink, respectively.

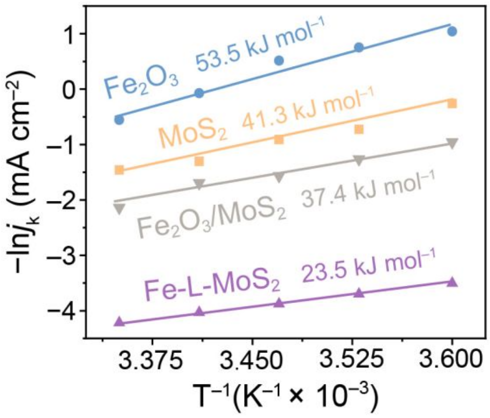

Figure S28. Arrhenius plots of MoS_2, Fe_2O_3, Fe_2O_3/MoS_2, and Fe-L-MoS_2.

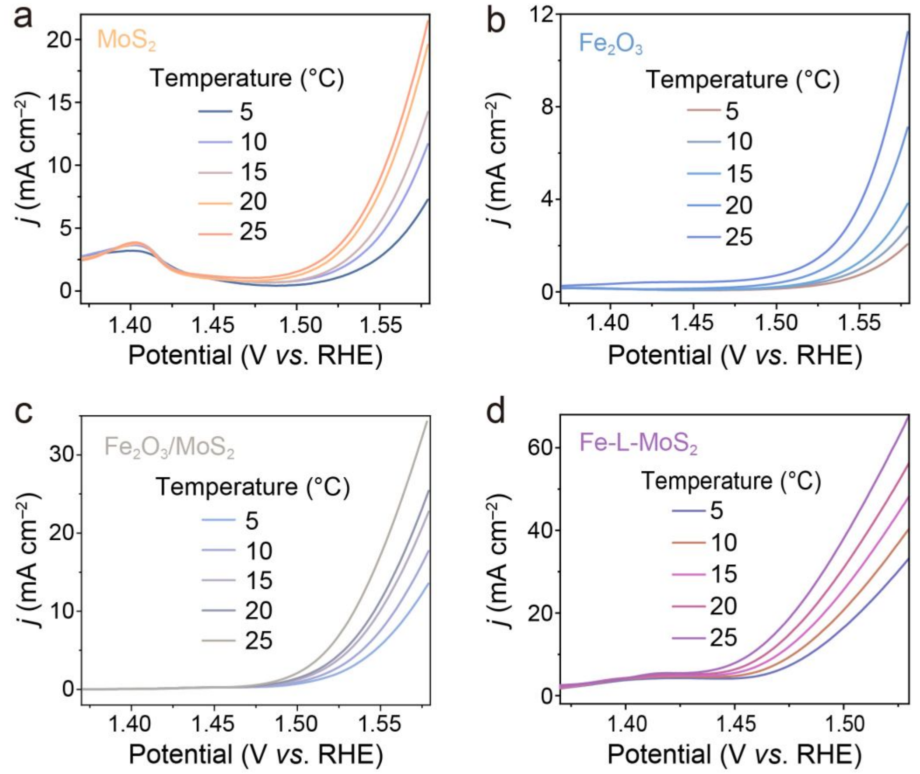

Figure S29. LSVs measured at various temperatures: (a) MoS_2, (b) Fe_2O_3, (c) Fe_2O_3/MoS_2, and (d) Fe-L-MoS_2.

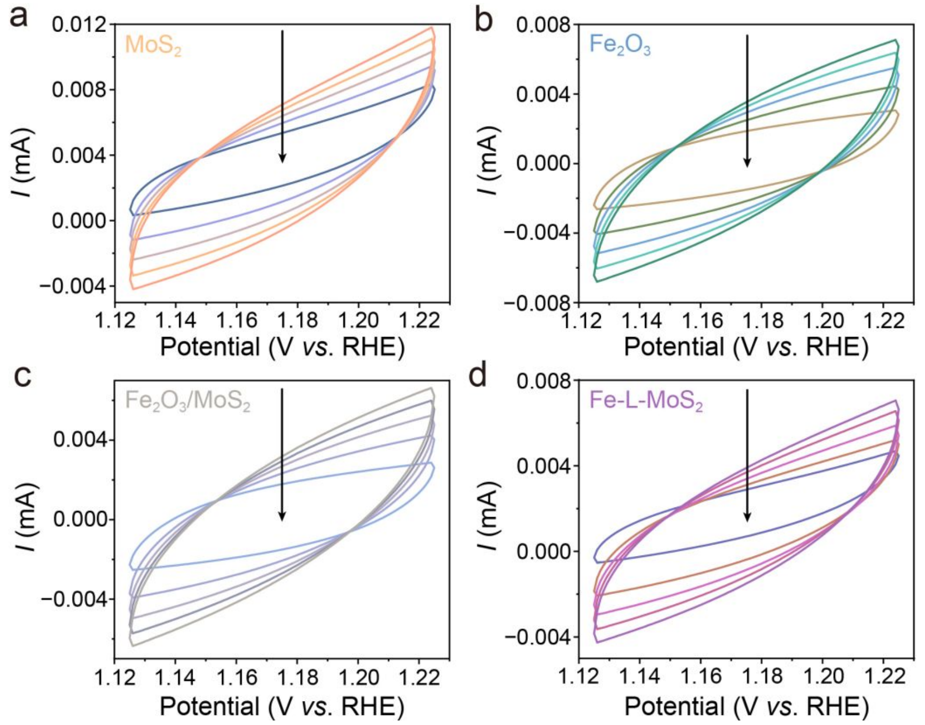

Figure S30. CV measured in a non-Faradaic region at various scan rates. (a) MoS_2, (b) Fe_2O_3, (c) Fe_2O_3/MoS_2, and (d) Fe-L-MoS_2. The scan rate is from 100 to 20 mV s^{-1}.

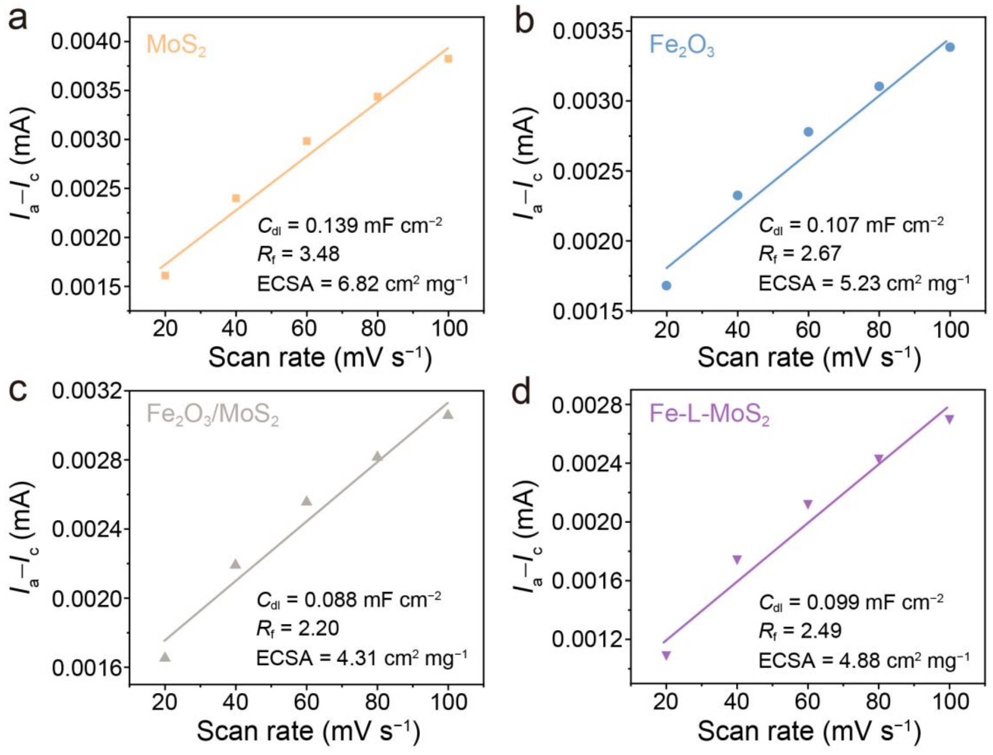

Figure S31. Linear fitting of double-layer capacitance (C_{dl}) *vs.* CV scan rate for the estimation of the ECSA of (a) MoS_2, (b) Fe_2O_3, (c) Fe_2O_3/MoS_2, and (d) Fe-L-MoS_2.

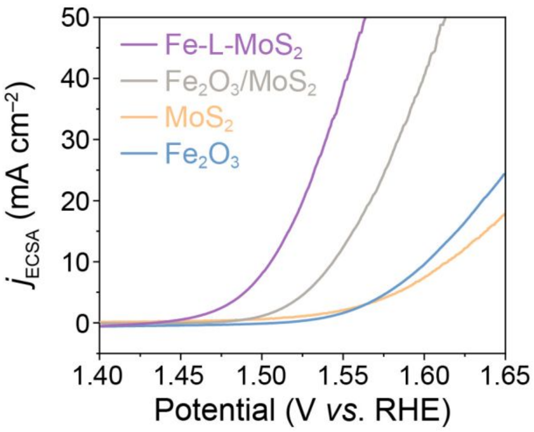

Figure S32. ECSA-normalized LSV curves of Fe_2O_3, MoS_2, Fe_2O_3/MoS_2, and Fe-L-MoS_2.

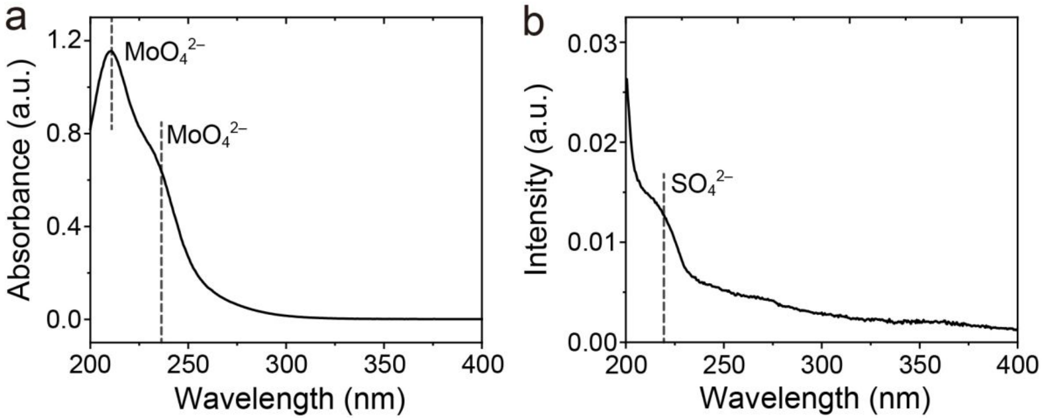

Figure S33. UV−vis absorption spectra of (a) 0.1 mM NH_4MoO_4 and (b) 0.01 mM Na_2SO_4.

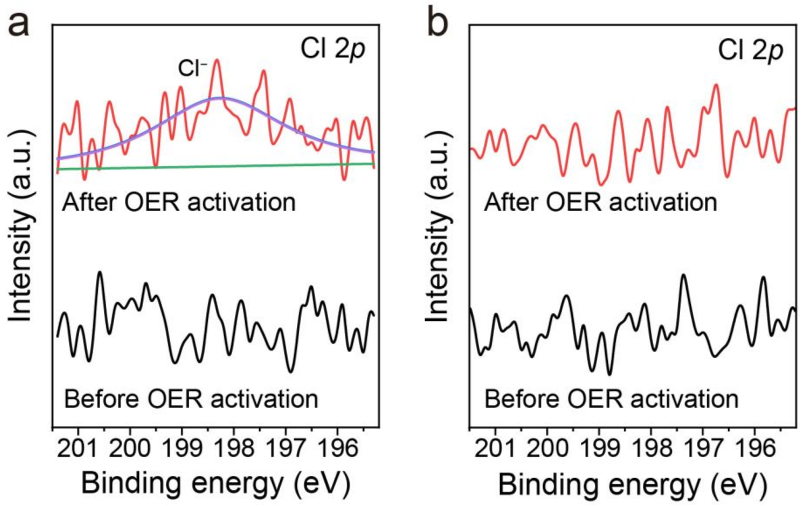

Figure S34. XPS Cl 2*p* spectra of (a) Fe_2O_3 and (b) Fe-L-MoS_2 before and after OER activation in saline water (0.5 M NaCl + 1.0 M KOH).

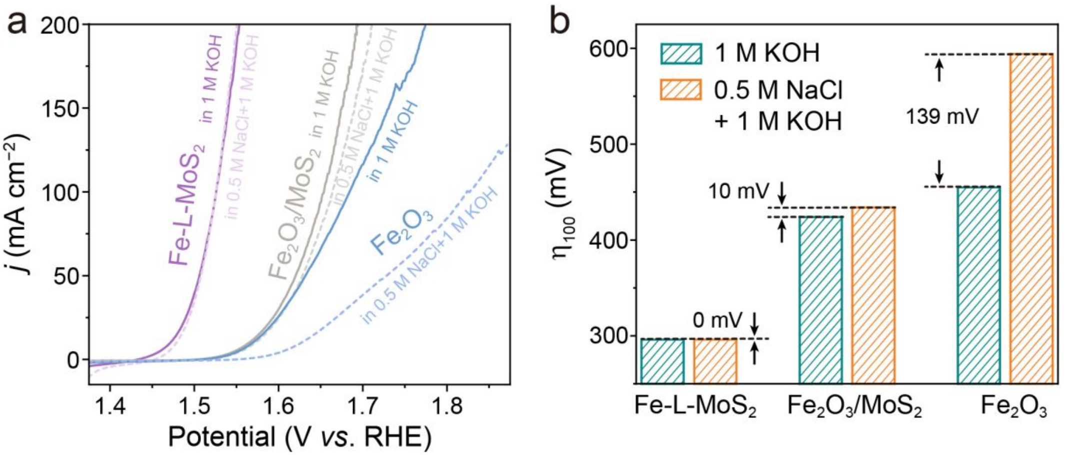

Figure S35. (a) LSV curves of Fe-L-MoS_2, Fe_2O_3/MoS_2, and Fe_2O_3 in alkaline saline water and seawater. (b) Comparison of OER overpotentials of Fe-L-MoS_2, Fe_2O_3/MoS_2, and Fe_2O_3 in various electrolytes.

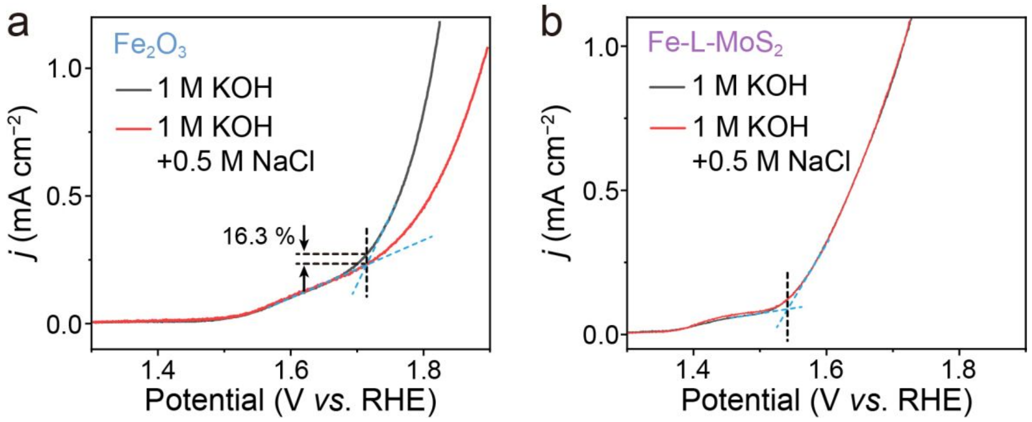

Figure S36. LSV curves of Fe_2O_3 and Fe-L-MoS_2 in different electrolytes on GCE without *i*R correction.

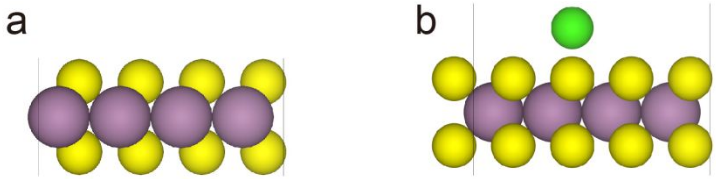

Figure S37. Structures of (a) MoS_2 and (b) *Cl-adsorbed MoS_2 (on a S site). Mo, O, S, and Cl atoms are shown in violet, red, yellow, and green, respectively.

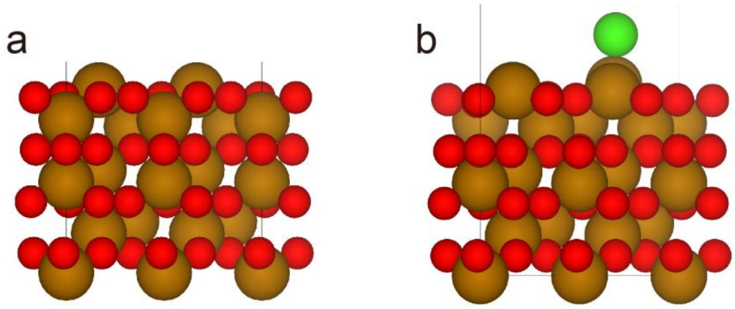

Figure S38. Structures of (a) Fe_2O_3 and (b) *Cl-adsorbed Fe_2O_3 (on a Fe site). Fe, O, and Cl atoms are shown in brown, red, and green, respectively.

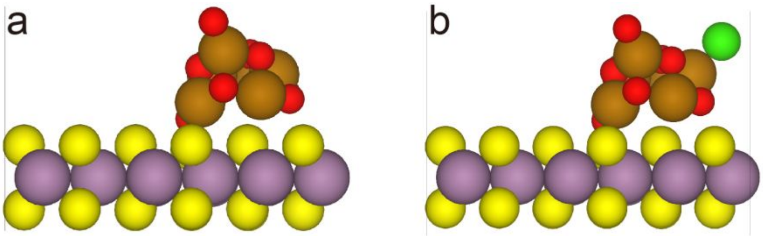

Figure S39. Structures of (a) Fe_2O_3/MoS_2 heterojunction and (b) *Cl-adsorbed Fe_2O_3/MoS_2 (on a Fe site). Mo, Fe, O, S, Cl, and H atoms are shown in violet, brown, red, yellow, green, and pink, respectively.

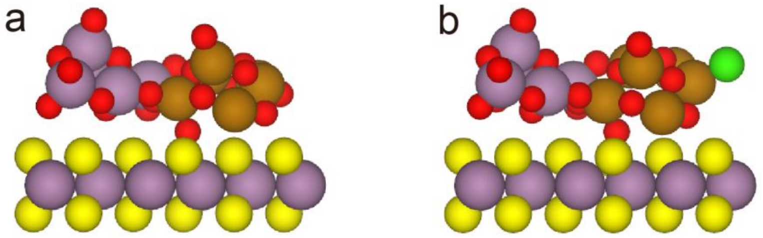

Figure S40. Structures of (a) $MoO_3/Fe_2O_3/MoS_2$ heterojunction and (b) *Cl-adsorbed $MoO_3/Fe_2O_3/MoS_2$ (on a Fe site). Mo, Fe, O, S, Cl, and H atoms are shown in violet, brown, red, yellow, green, and pink, respectively.

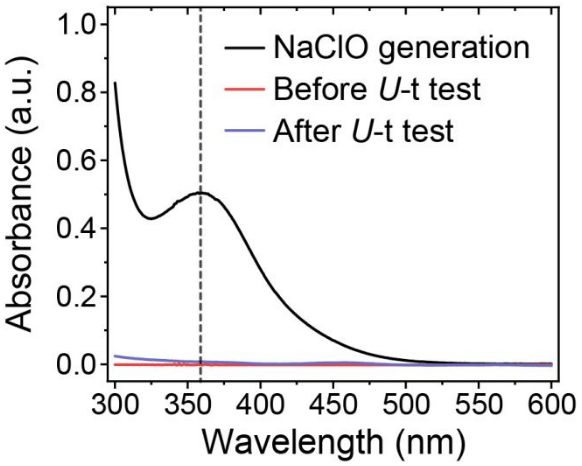

Figure S41. UV–vis spectra of electrolytes before and after the U-t test at 300 mA cm^{-2} for 500 h.

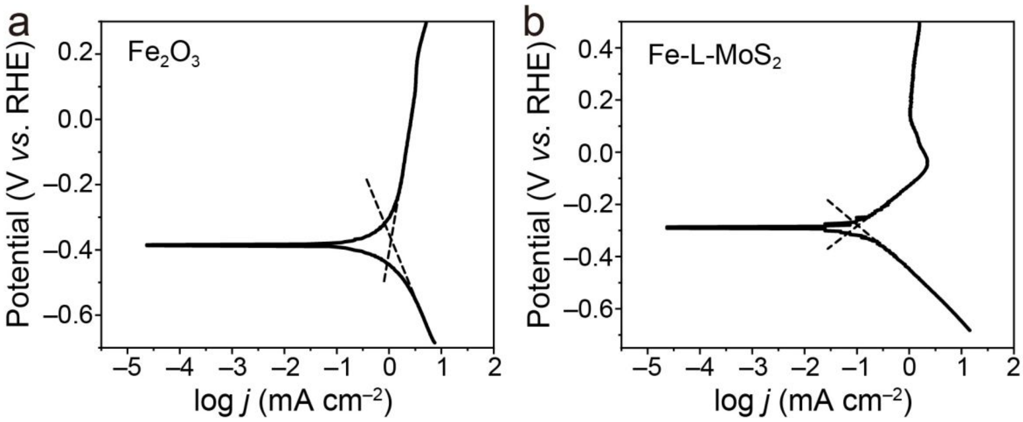

Figure S42. Corrosion polarization curves of (a) Fe_2O_3 and (b) Fe-L-MoS_2.

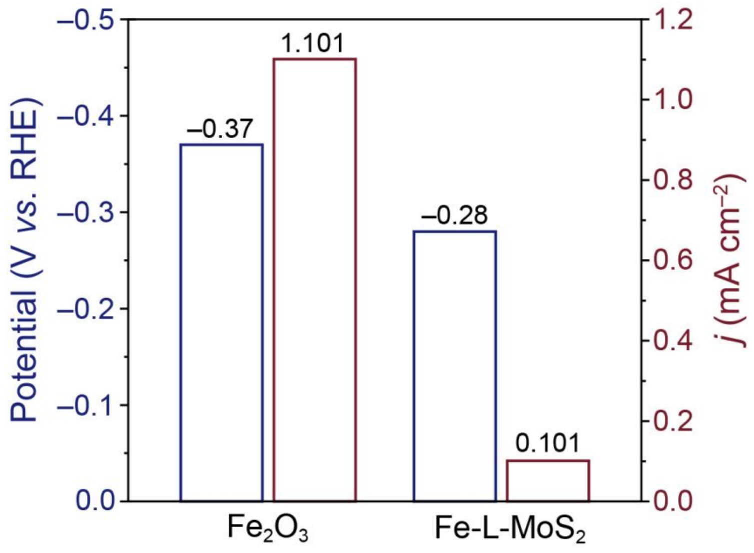

Figure S43. Corrosion current densities and potentials of pristine Fe_2O_3 and Fe-L-MoS_2 in natural seawater.

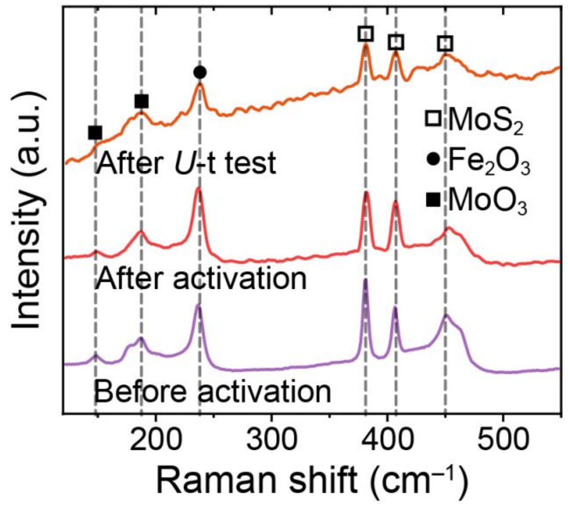

Figure S44. Raman spectra of Fe-L-MoS_2 before and after OER activation and after long-term *U*-t test in alkaline seawater.

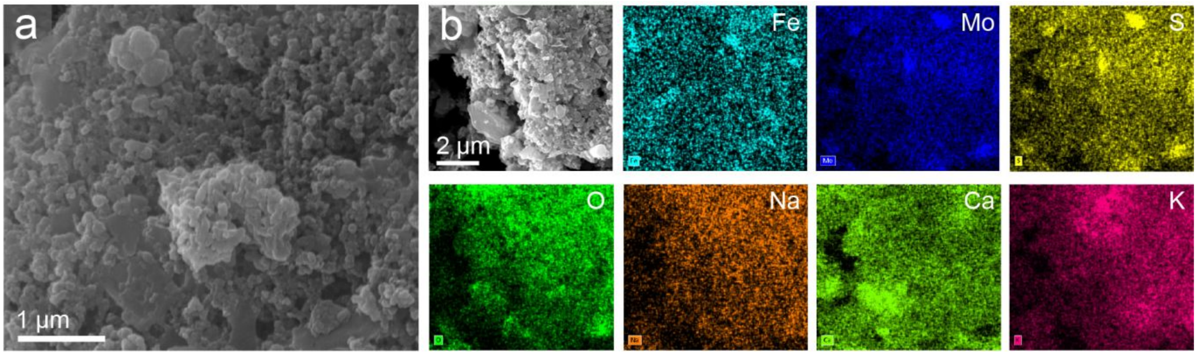

Figure S45. (a) SEM image and (b) energy-dispersive spectroscopic mapping images of Fe-L-MoS_2 after the OER stability test in seawater.

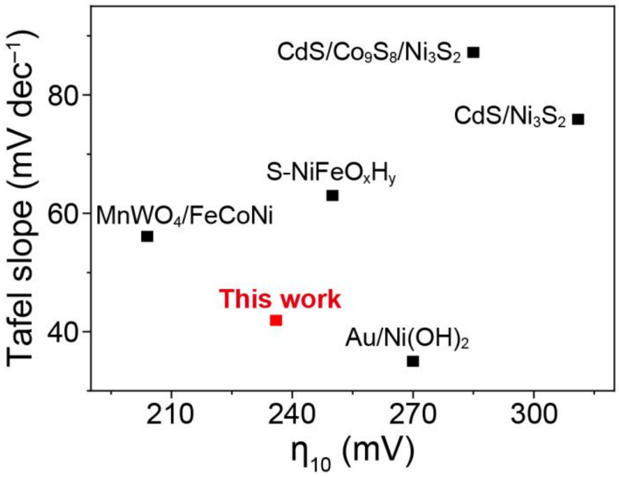

Figure S46. Comparison of OER overpotentials (η_{10}) and Tafel slopes for Fe-L-MoS_2 and previously reported electrocatalysts under light-assisted conditions.

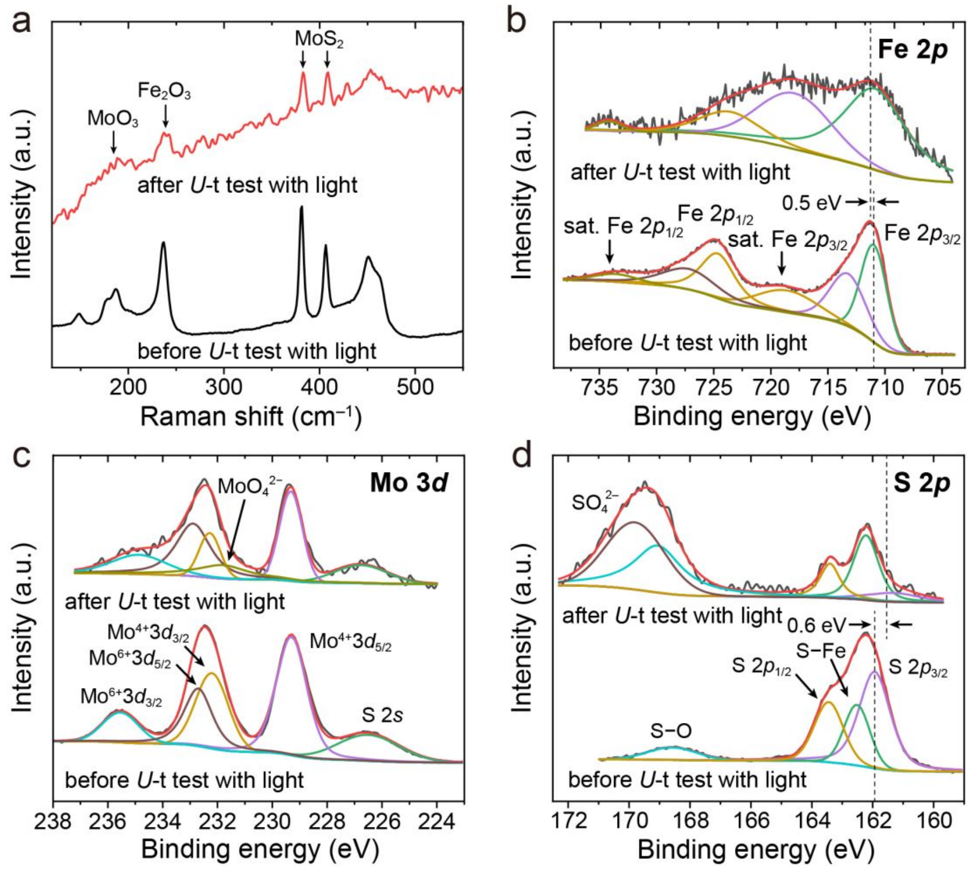

Figure S47. (a) Raman spectra and XPS (b) Fe 2*p*, (c) Mo 3*d*, and (d) S 2*p* spectra of Fe-L-MoS_2 before and after *U*-t test under light irradiation.

Following the long-term light-assisted *U*-t test, Raman spectra of Fe-L-MoS_2 (**Figure S47a**) exhibit a slight intensity reduction but retain characteristic MoO_3, Fe_2O_3, and MoS_2 peaks, indicating structural stability. XPS analysis reveals a 0.5-eV shift in Fe 2*p* peaks toward higher binding energy (**Figure S47b**), suggesting a minor increase in Fe valence. The presence of MoO_4^{2-} and SO_4^{2-} anions, detected in Mo 3*d* and S 2*p* spectra (**Figures S47c** and **S47d**), confirms their stability post-test, underscoring the robust anticorrosion properties of the $MoO_3/Fe_2O_3/MoS_2$ heterojunction.

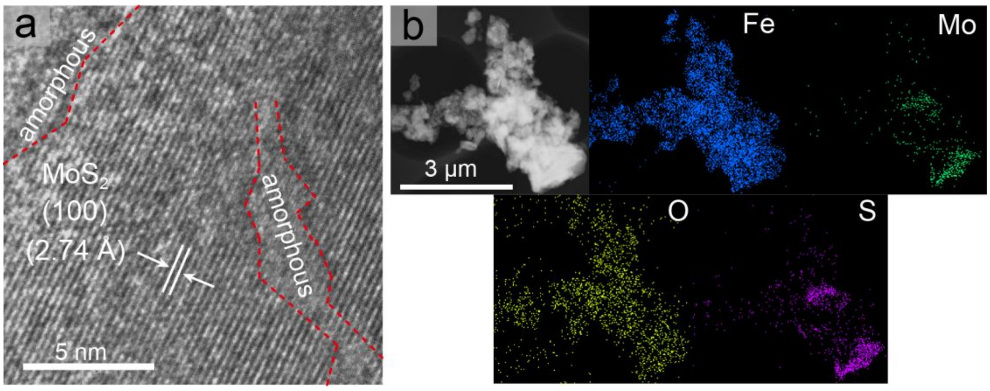

Figure S48. (a) High-resolution TEM and (b) STEM images with the corresponding EDS mapping images of Fe-L-MoS_2 after light-assisted *U*-t test.

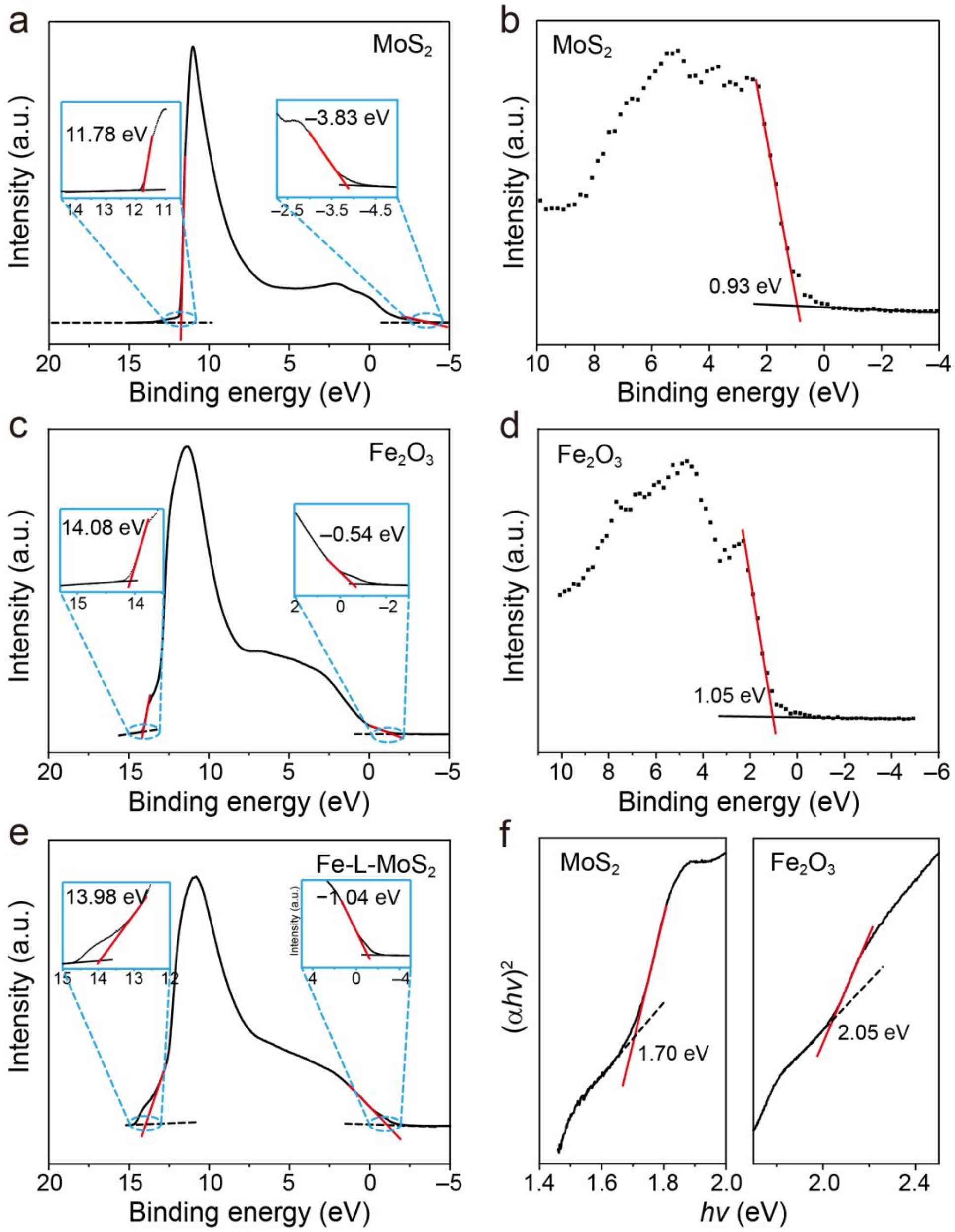

Figure S49. UPS spectra of (a) MoS_2, (c) Fe_2O_3, and (e) Fe-L-MoS_2. VB-XPS spectra of (b) MoS_2 and (d) Fe_2O_3. (f) Tauc plots of MoS_2 and Fe_2O_3.

Conduction band energy (E_{CB}), valence band energy (E_{VB}), and work function (Φ) were determined using UPS and XPS-VB spectra. Specifically, UPS spectra of MoS_2, Fe_2O_3, MoO_3, and Fe-L-MoS_2 were collected using a He Iα as the UV source (hv = 21.22 eV)[19, 20] and used to determine their work functions using the following equation:

$$\Phi = hv - E_{cutoff} + E_F \tag{24}$$

where E_{cutoff} and E_F are the binding energies of the secondary electron cutoff edge and Fermi level edge, respectively. **Figure S49a** indicates that the E_{cutoff} and E_F of MoS_2 are 11.78 and −3.83 eV, respectively, and thus the work function of MoS_2 was determined to be 5.62 eV. The

valence band maximum (VBM) of MoS_2 was estimated using the XPS-VB spectrum. The energy gap between the Fermi level and VBM is 0.93 eV (**Figure S49b**).[21, 22] Therefore, the energy level of VBM in MoS_2 is calculated to be 6.55 eV. Similarly, the UPS and XPS-VB spectra of Fe_2O_3 shown in **Figures S49c** and **S49d** were used to determine Φ/VBM relative to the vacuum level as 6.60/7.65. The Φ of Fe-L-MoS_2 relative to the vacuum level was calculated to be 6.20 eV (**Figure S49e**). Bandgap energy (E_g) was calculated using the Tauc plots. As shown in **Figure S49f**, the E_g values of MoS_2 and Fe_2O_3 were determined to be 1.70 and 2.05 eV, respectively. Subsequently, the E_{CB} values of MoS_2 and Fe_2O_3 were calculated as 4.85 and 5.60 eV according to the following equation:

$$E_{CB} = E_{VB} - E_g \quad (25)$$

The work function, E_{CB}, and E_{VB} of MoO_3 are 5.1, 4.8, and 7.8 eV, respectively.[23]

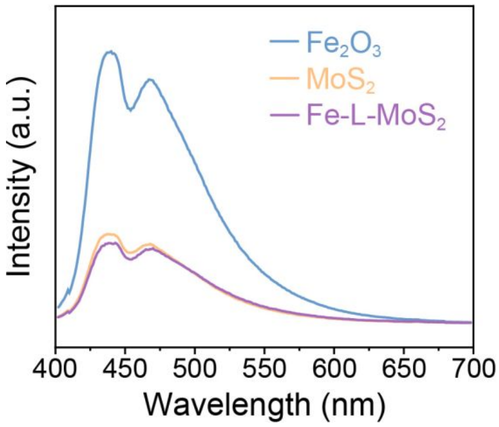

Figure S50. PL spectra of Fe_2O_3, MoS_2, and Fe-L-MoS_2.

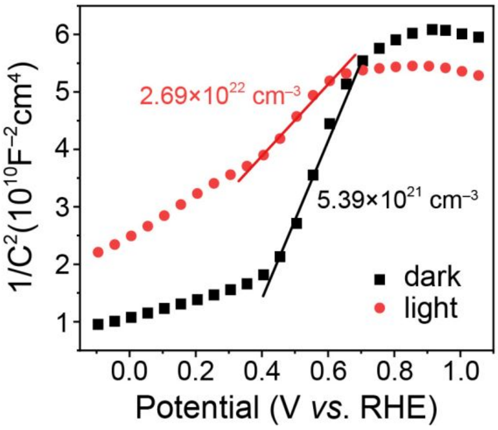

Figure S51. Mott–Schottky plots of Fe-L-MoS_2 in dark and under light irradiation.

3. Supplementary Tables

Table S1. BET surface area of MoS_2 samples.

Sample	BET surface area (m^2 g^{-2})
Pristine MoS_2 powder	8.33
Fe-L-MoS_2-5 min	15.82
Fe-L-MoS_2-15 min	38.63
Fe-L-MoS_2-25 min	47.35
Fe-L-MoS_2-35 min	50.06

Table S2. ICP-OES analysis of metals in Fe-L-MoS_2 samples prepared using various laser ablation durations (with [$Fe(NO_3)_3$] = 0.5 M) and $Fe(NO_3)_3$ concentrations (with 25-min laser ablation).

Sample	Element content (ppm)		Fe/Mo Atomic ratio
	Mo	Fe	
MoS_2	44.79	0	-
Fe_2O_3/MoS_2	13.31	15.15	1.94
Fe-L-MoS_2-5 min	33.71	0.82	0.04
Fe-L-MoS_2-15 min	26.88	6.03	0.39
Fe-L-MoS_2-25 min	19.63	11.86	1.04
Fe-L-MoS_2-35 min	13.63	15.37	1.94
0.025 M Fe-L-MoS_2	29.16	6.93	0.41
0.1 M Fe-L-MoS_2	15.57	14.22	1.56

Table S3. Mole ratios of Fe_2O_3 and Mo^{6+}/Mo^{4+} atomic ratio in Fe-L-MoS_2 samples prepared using various laser ablation durations.

Sample	Fe_2O_3 Concentration (mol %)	Mo^{6+}/Mo^{4+} Atomic ratio
Fe-L-MoS_2-15 min	16.1	0.35
Fe-L-MoS_2-25 min	34.2	0.48
Fe-L-MoS_2-35 min	49.2	0.57

Table S4. Comparison of the OER performance of Fe-L-MoS_2 with previously reported MoS_2-based electrocatalysts (in 1 M KOH).

Sample	Overpotential at 10 mA cm^{-2} (η_{10}, mV)	Tafel slope (mV dec^{-1})	Ref.
Fe-L-MoS_2	241	48.9	**This work**
CeO_2@CoS/MoS_2	247	64	24
N-doped carbon nanotube@CoP@MoS_2	234	81	25
MCNTs@CoS_x@MoS_2	285	76	26
Co,Nb-MoS_2/TiO_2	260	59	27
O-MoS_2@Pt	244	53	28
Co–Ni–P/MoS_2	235	71	29
Co–Ru-MoS_2	308	50	30
(Ni, Fe)S_2@MoS_2	270	43	31
Ni_3S_2@MoS_2/FeOOH	234	49	32
vacancy-rich MoS_2/NPF-$CoFe_2O_4$	250	41	33
FeCoNiMnCr	261	42.2	34
$NiFeCoCrW_{0.2}$	220	36.8	35
Fe_2O_3-$NiFe_2O_4$	266	41.8	36
Fe-Co$(OH)_2$/Fe_2O_3	219	32.6	37
Ru-Fe_2O_3/CoS	264	61.9	38

Table S5. Parameters of EIS simulation for catalysts of MoS_2, Fe_2O_3, Fe_2O_3/MoS_2, and Fe-L-MoS_2.

Deposition time (min)	R_s (Ω)	R_{ct} (Ω)	R_2 (Ω)	CPE_1 (F)	CPE_2 (F)	W (S sec^5)
MoS_2	1.45	74.67	0.61	0.011	0.007	0.054
Fe_2O_3	1.43	114.50	3.30	0.005	0.001	0.035
Fe_2O_3/MoS_2	1.45	3.47	1.26	0.002	0.001	0.836
Fe-L-MoS_2	1.41	2.00	0.24	0.005	0.221	0.161

Table S6. Comparison of OER performance of Fe-L-MoS_2 with previously reported light-assisted OER electrocatalysts (in 1 M KOH).

Sample	η_{10} (mV), dark (light)	Tafel slope (mV dec^{-1}), dark (light)	Ref.
Fe-L-MoS_2	241(236)	48.9(41.9)	**This work**
$MnWO_4$/FeCoNi	234(204)	67.4(56.1)	39
S-$NiFeO_xH_y$	265(250)	78(63)	40
Au/$Ni(OH)_2$	330(270)	43(35)	41
CdS/Co_9S_8/Ni_3S_2	310(285)	91.3(87.2)	42
CdS/Ni_3S_2	330(311)	85.8(75.9)	43

Table S7. Lifetimes of carriers in Fe_2O_3, MoS_2, and Fe-L-MoS_2.

Sample	τ_1			τ_2		
	Value (ns)	A_1	Rel.%	Value (ns)	A_2	Rel.%
Fe_2O_3	0.40	200.57	66.08	2.69	15.38	33.92
MoS_2	0.60	151.54	43.40	2.94	40.54	56.60
Fe-L-MoS_2	1.07	181.94	31.56	4.18	101.02	68.44

4. References

(1) Shi, L.; Li, Y.; Zeng, F. L.; Ran, S. J.; Dong, C. Y.; Leu, S. Y.; Boles, S. T.; Lam, K. H. In situ growth of amorphous Fe_2O_3 on 3D interconnected nitrogen-doped carbon nanofibers as high-performance anode materials for sodium-ion batteries. *Chem. Eng. J.* **2019**, *356*, 107−116.

(2) Huang, C. Q.; Zhou, Q. C.; Yu, L.; Duan, D. S.; Cao, T. Y.; Qiu, S. H.; Wang, Z. Z.; Guo, J.; Xie, Y. X.; Li, L. P.; et al. Functional bimetal co-modification for boosting large-current-density seawater electrolysis by inhibiting adsorption of chloride ions. *Adv. Energy Mater.* **2023**, *13* (32), 2301475.

(3) Kim, M.; Anjum, M. A. R.; Lee, M.; Lee, B. J.; Lee, J. S. Activating MoS_2 basal plane with Ni_2P nanoparticles for Pt-like hydrogen evolution reaction in acidic media. *Adv. Funct. Mater.* **2019**, *29* (10), 1809151.

(4) Kresse, G.; Furthmuller, J. Efficient iterative schemes for ab initio total-energy calculations using a plane-wave basis set. *Phys. Rev. B* **1996**, *54* (16), 11169−11186.

(5) Kresse, G.; Hafner, J. Ab initio molecular-dynamics simulation of the liquid-metal–amorphous-semiconductor transition in germanium. *Phys. Rev. B* **1994**, *49* (20), 14251−14269.

(6) Kresse, G.; Hafner, J. Ab initio molecular dynamics for liquid metals. *Phys. Rev. B* **1993**, *47* (1), 558−561.

(7) Kresse, G.; Joubert, D. From ultrasoft pseudopotentials to the projector augmented-wave method. *Phys. Rev. B* **1999**, *59* (3), 1758−1775.

(8) Blochl, P. E. Projector augmented-wave method. *Phys. Rev. B* **1994**, *50* (24), 17953−17979.

(9) Perdew, J. P.; Burke, K.; Ernzerhof, M. Generalized gradient approximation made simple. *Phys. Rev. Lett.* **1997**, *78* (7), 1396−1396.

(10) Grimme, S.; Antony, J.; Ehrlich, S.; Krieg, H. A consistent and accurate ab initio parametrization of density functional dispersion correction (DFT-D) for the 94 elements H-Pu. *J. Chem. Phys.* **2010**, *132* (15), 154104.

(11) Dudarev, S. L.; Botton, G. A.; Savrasov, S. Y.; Humphreys, C. J.; Sutton, A. P. Electron-energy-loss spectra and the structural stability of nickel oxide: An LSDA+U study. *Phys. Rev. B* **1998**, *57* (3), 1505−1509.

(12) Car, R.; Parrinello, M. Unified approach for molecular dynamics and density-functional theory. *Phys. Rev. Lett.* **1985**, *55* (22), 2471−2474.

(13) Tang, W.; Sanville, E.; Henkelman, G. A grid-based Bader analysis algorithm without lattice bias. *J. Phys.: Condens. Matter* **2009**, *21* (8), 084204.

(14) Sattari-Esfahlan, S. M.; Kim, H. G.; Hyun, S. H.; Choi, J. H.; Hwang, H. S.; Kim, E. T.; Park, H. G.; Lee, J. H. Low-temperature direct growth of amorphous boron nitride films for high-performance nanoelectronic device applications. *ACS Appl. Mater. Inter.* **2023**, *15*, 7274−7281.

(15) Liu, H. Q.; Yao, C. B.; Liu, X. J.; Jiang, C. H. Heterointerface-enhanced ultrafast carrier dynamics and nonlinear optical response via constructing electronic structure-induced type-I ZnO-MoS_2 n-n heterojunction. *Appl. Surf. Sci.* **2022**, *580*, 152222.

(16) Narengerile, T. W. Acetone decomposition by water plasmas at atmospheric pressure. *Chem. Eng. Sci.* **2012**, *69* (1), 296−303.

(17) Fiedor, J. N.; Proctor, A.; Houalla, M.; Sherwood, P. M. A.; Mulcahy, F. M.; Hercules, D. M. Use of ESCA valence bands to infer structural information about the molybdenum phase in supported molybdenum catalysts. *J. Phys. Chem.* **1992**, *96* (26), 10967−10970.

(18) Zhang, Z.; Wang, M.; Zhou, H. R.; Wang, F. Surface sulfate ion on CdS catalyst enhances syngas generation from biopolyols. *J. Am. Chem. Soc.* **2021**, *143* (17), 6533−6541.

(19) Gao, C.; Meng, Q. Q.; Zhao, K.; Yin, H. J.; Wang, D. W.; Guo, J.; Zhao, S. L.; Chang, L.; He, M.; Li, Q. X.; et al. Co_3O_4 hexagonal platelets with controllable facets enabling highly efficient visible-light photocatalytic reduction of CO_2. *Adv. Mater.* **2016**, *28* (30), 6485−6490.
(20) Greiner, M. T.; Helander, M. G.; Tang, W. M.; Wang, Z. B.; Qiu, J.; Lu, Z. H. Universal energy-level alignment of molecules on metal oxides. *Nat. Mater.* **2012**, *11* (1), 76−81.
(21) Chen, X. B.; Liu, L.; Yu, P. Y.; Mao, S. S. Increasing solar absorption for photocatalysis with black hydrogenated titanium dioxide nanocrystals. *Science* **2011**, *331* (6018), 746−750.
(22) Hermans, Y.; Klein, A.; Sarker, H. P.; Huda, M. N.; Junge, H.; Toupance, T.; Jaegermann, W. Pinning of the fermi level in $CuFeO_2$ by polaron formation limiting the photovoltage for photochemical water splitting. *Adv. Funct. Mater.* **2020**, *30* (10), 1910432.
(23) Liu, W.; Tian, Q. Y.; Yang, J.; Zhou, Y. N.; Chang, H. W.; Cui, W. H.; Xu, Q. A two-dimensional amorphous plasmonic heterostructure of Pd/MoO_{3-x} for enhanced photoelectrochemical water splitting performance. *Chem. Asian J.* **2021**, *16* (10), 1253−1257.
(24) Huang, W. H.; Li, X. M.; Yang, X. F.; Zhang, H. Y.; Liu, P. B.; Ma, Y. M.; Lu, X. CeO_2-embedded mesoporous CoS/MoS_2 as highly efficient and robust oxygen evolution electrocatalyst. *Chem. Eng. J.* **2021**, *420*, 127595.
(25) Zhang, C. A. L.; Xie, Y.; Liu, J. T.; Cao, F. H.; Cong, H. P.; Li, H. 1D core-shell MOFs derived CoP nanoparticles-embedded N-doped porous carbon nanotubes anchored with MoS_2 nanosheets as efficient bifunctional electrocatalysts. *Chem. Eng. J.* **2021**, *419*, 129977.
(26) Wang, C.; Zhang, L.; Xu, G. C.; Yang, L. F.; Yang, J. H. Construction of unique ternary composite MCNTs@CoSx@MoS_2 with three-dimensional lamellar heterostructure as high-performance bifunctional electrocatalysts for hydrogen evolution and oxygen evolution reactions. *Chem. Eng. J.* **2021**, *417*, 129270.
(27) Nguyen, D. C.; Doan, T. L. L.; Prabhakaran, S.; Tran, D. T.; Kim, D.; Lee, J. H.; Kim, N. H. Hierarchical Co and Nb dual-doped MoS_2 nanosheets shelled micro-TiO_2 hollow spheres as effective multifunctional electrocatalysts for HER, OER, and ORR. *Nano Energy* **2021**, *82*, 105750.
(28) Gong, F. L.; Ye, S.; Liu, M. M.; Zhang, J. W.; Gong, L. H.; Zeng, G.; Meng, E. C.; Su, P. P.; Xie, K. F.; Zhang, Y. H.; et al. Boosting electrochemical oxygen evolution over yolk-shell structured O-MoS_2 nanoreactors with sulfur vacancy and decorated Pt nanoparticles. *Nano Energy* **2020**, *78*, 105284.
(29) Bao, J. H.; Zhou, Y. M.; Zhang, Y. W.; Sheng, X. L.; Wang, Y. Y.; Liang, S.; Guo, C.; Yang, W.; Zhuang, T.; Hu, Y. J. Engineering water splitting sites in three-dimensional flower-like Co-Ni-P/MoS_2 heterostructural hybrid spheres for accelerating electrocatalytic oxygen and hydrogen evolution. *J. Mater. Chem. A* **2020**, *8* (42), 22181−22190.
(30) Kwon, I. S.; Debela, T. T.; Kwak, I. H.; Park, Y. C.; Seo, J.; Shim, J. Y.; Yoo, S. J.; Kim, J. G.; Park, J.; Kang, H. S. Ruthenium nanoparticles on cobalt-doped 1T phase MoS_2 nanosheets for overall water splitting. *Small* **2020**, *16* (13), 2000081.
(31) Liu, Y. K.; Jiang, S.; Li, S. J.; Zhou, L.; Li, Z. H.; Li, J. M.; Shao, M. F. Interface engineering of (Ni, Fe)S_2@MoS_2 heterostructures for synergetic electrochemical water splitting. *Appl. Catal. B-Environ. Energy* **2019**, *247*, 107−114.
(32) Zheng, M. Y.; Guo, K. L.; Jiang, W. J.; Tang, T.; Wang, X. Y.; Zhou, P. P.; Du, J.; Zhao, Y. Q.; Xu, C. L.; Hu, J. S. When MoS_2 meets FeOOH: A "one-stone-two-birds" heterostructure as a bifunctional electrocatalyst for efficient alkaline water splitting. *Appl. Catal. B-Environ. Energy* **2019**, *244*, 1004−1012.

(33) Sun, J.; Guo, N. K.; Shao, Z. Y.; Huang, K. K.; Li, Y. W.; He, F.; Wang, Q. A facile strategy to construct amorphous spinel-based electrocatalysts with massive oxygen vacancies using ionic liquid dopant. *Adv. Energy Mater.* **2018**, *8* (27), 1800980.
(34) Hu, J.; Guo, T. Q.; Zhong, X. Y.; Li, J.; Mei, Y. J.; Zhang, C. X.; Feng, Y. B.; Sun, M. Z.; Meng, L. J.; Wang, Z. Y.; et al. In situ reconstruction of high-entropy heterostructure catalysts for stable oxygen evolution electrocatalysis under industrial conditions. *Adv. Mater.* **2024**, *36* (14), 2310918.
(35) Zhang, T.; Zhao, H. F.; Chen, Z. J.; Yang, Q.; Gao, N.; Li, L.; Luo, N.; Zheng, J.; Bao, S. D.; Peng, J.; et al. High-entropy alloy enables multi-path electron synergism and lattice oxygen activation for enhanced oxygen evolution activity. *Nat. Commun.* **2025**, *16* (1), 3327.
(36) Li, Y.; Zhang, Y.; Shi, R.; Xu, X.; Wang, X.; Zhou, G. Hollow Fe_2O_3-$NiFe_2O_4$ hetero-nanoframes coupled with N-doped graphene for boosted electrochemical water oxidation through optimizing intermediate bonding. *Chem. Eng. J.* **2025**, *512*, 162666.
(37) Xin, S. S.; Tang, Y.; Jia, B. H.; Zhang, Z. F.; Li, C. P.; Bao, R.; Li, C. J.; Yi, J. H.; Wang, J. S.; Ma, T. Y. Coupling adsorbed evolution and lattice oxygen mechanism in Fe-$Co(OH)_2/Fe_2O_3$ heterostructure for enhanced electrochemical water oxidation. *Adv. Funct. Mater.* **2023**, *33* (45), 2305243.
(38) Chen, X.; Kong, Y. L.; Yin, H. F.; Yang, X. Y.; Zhao, Q. Y.; Xiao, D. D.; Wang, Z. L.; Zhang, Y. Z.; Xue, Q. K. Unveiling the enhancement of electrocatalytic oxygen evolution activity in Ru-Fe_2O_3/CoS heterojunction catalysts. *Small* **2024**, *20* (46), 2403427.
(39) Jiang, M. M.; Xu, J.; Chen, Y. J.; Wang, L. Q.; Munroe, P.; Xie, Z. H.; Peng, S. J. High-efficiency photo-assisted large current-density water splitting with Mott-Schottky heterojunctions. *Angew. Chem. Int. Ed.* **2025**, *64* (3), e202415492.
(40) Zhang, Y. J.; Song, X. J.; Xue, S.; Liang, Y. G.; Jiang, H. Q. Fabrication of hierarchically structured S-doped NiFe hydroxide/oxide electrodes for solar-assisted oxygen evolution reaction in seawater splitting. *Applied Catalysis a-General* **2023**, *649*, 118965.
(41) Liu, G. G.; Li, P.; Zhao, G. X.; Wang, X.; Kong, J. T.; Liu, H. M.; Zhang, H. B.; Chang, K.; Meng, X. G.; Kako, T.; et al. Promoting active species generation by plasmon-induced hot-electron excitation for efficient electrocatalytic oxygen evolution. *J. Am. Chem. Soc.* **2016**, *138* (29), 9128−9136.
(42) Si, F. Y.; Tang, C. Y.; Gao, Q. Z.; Peng, F.; Zhang, S. S.; Fang, Y. P.; Yang, S. Y. Bifunctional CdS@Co_9S_8/Ni_3S_2 catalyst for efficient electrocatalytic and photo-assisted electrocatalytic overall water splitting. *J. Mater. Chem. A* **2020**, *8* (6), 3083−3096.
(43) Yang, S. Y.; Guan, H. J.; Zhong, Y. M.; Quan, J. X.; Luo, N.; Gao, Q. Z.; Xu, Y. H.; Peng, F.; Zhang, S. S.; Fang, Y. P. CdS@Ni_3S_2 for efficient and stable photo-assisted electrochemical (P-EC) overall water splitting. *Chem. Eng. J.* **2021**, *405*, 126231.
